# Multimodal Plant–Animal Communication: Signaling Mechanisms, Coevolution, and Environmental Disruption

**DOI:** 10.3390/plants15192960

**Published:** 2026-09-28

**Authors:** Hiba A. Jasim, Marwa Y. Khudair, Sura A. Jasim, Salwan M. Abdulateef

**Affiliations:** 1 Department of Biology, College of Education for Pure Sciences, University of Anbar, Ramadi 31001, Iraq; h.a.jasim@uoanbar.edu.iq; 2 Department of Soil Sciences and Water Resources, College of Agriculture, University of Anbar, Ramadi 31001, Iraq; ag.marwa.yass@uoanbar.edu.iq; 3 Habbaniyah District, Anbar Governorate, Habbaniyah 31010, Iraq; abbas_sura@yahoo.com; 4 Department of Animal Production, College of Agriculture, University of Anbar, Ramadi 31001, Iraq

**Keywords:** plant signaling, plant–animal interactions, multimodal signaling, volatile organic compounds, sensory ecology, coevolution, plant bioacoustics, climate change, biodiversity conservation, terrestrial ecosystems

## Abstract

Plant–animal communication involves diverse forms of information transfer that can mediate ecologically important interactions, including pollination, seed dispersal, herbivory, and plant defense. This review synthesizes current knowledge of the chemical, visual, gustatory, electrical, acoustic, and vibrational information involved in these interactions, with particular emphasis on volatile organic compounds, herbivore-derived elicitors and effectors, multimodal signaling, sensory perception, learning, and evolutionary processes, including coevolution where reciprocal evidence is available. A central distinction is made between detectable traits or cues and functional biological signals, emphasizing the need for evidence linking signal production or modification to receiver detection, behavioral or physiological responses, and ecologically meaningful outcomes. Acoustic and vibrational phenomena are considered critically because their communicative significance remains less firmly established than that of chemical and visual signaling. The review further examines how climate change and other anthropogenic pressures may alter signal production, transmission, perception, and phenological synchrony, thereby reorganizing plant–animal interactions. Genetic manipulation of hormonal, volatile, and specialized-metabolite pathways is also evaluated as a potential tool for modifying plant signaling, together with its possible unintended ecological consequences. By integrating plant physiology, sensory ecology, animal behavior, evolution, and environmental change, this review identifies major evidence gaps and highlights the importance of plant–animal communication for biodiversity conservation, pollination, biological control, and sustainable agriculture.

## 1. Introduction

Plant–animal interactions are fundamental to the structure and functioning of terrestrial ecosystems. They influence plant reproduction, animal foraging, community organization, and the maintenance of biodiversity through processes such as pollination, seed dispersal, herbivory, and plant defense [1,2]. These interactions depend not only on physical contact between organisms but also on the exchange of information. Plants produce a wide range of signals that can be detected and interpreted by animals, allowing them to locate food resources, recognize suitable hosts, avoid harmful plants, or identify reproductive opportunities. Floral and fruit traits, for example, can provide visual and olfactory information that guides animal behavior. Fluorescence has been proposed as a potential mechanism of colour-signal enhancement in several biological systems, including flowers, although its functional role in visually mediated behavior remains insufficiently demonstrated in many cases [3]. Fruit scent, by contrast, can function as an evolved cue associated with animal-mediated seed dispersal [4].

Communication between plants and animals is particularly complex because the interacting organisms differ fundamentally in their physiology, sensory systems, and modes of response. Plants lack a nervous system and centralized sensory organs, yet they can produce chemical, visual, gustatory, structural, electrical, and potentially acoustic cues that influence animal behavior [2]. Animals, in turn, possess sensory systems that allow them to detect and evaluate these cues according to their ecological requirements. The effectiveness of a plant signal therefore depends not only on its production but also on its detectability, reliability, and biological relevance to the receiver.

In this review, “information transfer” is used as the broadest term for cases in which a receiver obtains biologically relevant information from a plant- or animal-derived trait. A “cue” refers to information that can be used by a receiver without requiring that the trait evolved or is maintained for communication, whereas a “signal” is used more restrictively for a trait whose production or expression is shaped by its effects on receiver behavior and the resulting consequences for the signaler. “Chemical interaction” refers broadly to chemically mediated effects between organisms and does not necessarily imply communication, while a “plant response” denotes a physiological or molecular change that should not itself be classified as communication unless it forms part of a demonstrated information-transfer pathway [2,5,6]. This relationship is especially evident in pollination and seed-dispersal systems, where plant traits may evolve in response to the sensory capabilities and behavioral preferences of animal partners [1,4].

Plant–animal interactions often involve multiple sensory channels, and animals may integrate combinations of color, odor, shape, taste, and other traits during decision-making. These interactions can range from mutualistic to exploitative, as illustrated by nectar robbing [7]. Chemical signaling is one of the best-documented components of plant–animal communication. Plant volatile organic compounds can provide information associated with plant identity, resource availability, or physiological condition, and animals may use this information during host location, foraging, and other ecological decisions [2]. More detailed consideration of volatile blend composition, transmission, and receiver responses is provided in Section Plant Volatile Information in Host Location, Foraging, and Indirect Defense.

Visual and chemical signals are comparatively well established, whereas the possible role of acoustic and vibrational phenomena in plant biology remains an emerging area of research. Plants can generate sounds associated with physiological processes, and increasing attention has been directed toward whether such sounds have ecological functions [8]. Evidence is stronger for passive acoustic interactions in which plant structures modify or reflect animal-generated sounds. In some bat-pollinated systems, floral structures enhance echo-acoustic detectability and may help echolocating bats locate flowers [2]. However, the production of sound by plants should not automatically be interpreted as intentional communication. The distinction between physiological sound production, acoustic cues, and true signaling remains important and requires further experimental investigation [8].

Environmental change can further modify the effectiveness of plant–animal communication. Changes in temperature, water availability, atmospheric composition, and seasonal timing can alter plant phenology and the production of signals used by animal partners. Long-term drought, for example, can modify floral scent and influence pollinator visitation [9]. Climate-driven shifts in flowering phenology may also alter the temporal overlap between plants and their pollinators, potentially weakening previously established interactions [10]. These effects are important because successful communication depends on both the production of a detectable signal and the presence of an appropriate receiver at the correct place and time.

Despite substantial progress in chemical ecology, sensory ecology, plant defense, and behavioral ecology, these processes are often studied separately. As a result, the mechanisms linking signal production by plants, perception by animals, behavioral responses, and reciprocal evolutionary change remain incompletely integrated. This is particularly relevant for multimodal signaling, in which several sensory channels operate simultaneously, and for interactions in which signals can be exploited, manipulated, or disrupted by environmental conditions. A broader framework is therefore needed to connect the physiological mechanisms of signal production with animal perception, ecological outcomes, and evolutionary dynamics.

This review synthesizes current knowledge of multimodal communication between plants and animals, with particular emphasis on the mechanisms through which plants generate signals and animals detect and respond to them. It examines chemical, visual, gustatory, and acoustic components of communication; animal-derived signals that modify plant responses; sensory perception and behavioral decision-making; and the reciprocal evolutionary processes that shape signal reliability, exploitation, and coadaptation. The review also considers how environmental change may alter the production, transmission, and perception of these signals. By integrating ecological, physiological, sensory, and evolutionary perspectives, we aim to identify major gaps in current knowledge and highlight the relevance of plant–animal communication to biodiversity conservation, pollination, biological pest control, and sustainable agriculture. Throughout this review, coevolution is treated as a specific evolutionary process requiring evidence of reciprocal selective change rather than being inferred solely from correspondence between plant traits and animal sensory preferences. Accordingly, we distinguish reciprocal coevolution from related evolutionary processes and patterns, including coadaptation, diffuse coevolution, sensory bias, receiver exploitation, pollinator-mediated selection, phylogenetic correlation, and unidirectional or asymmetric selection.

### Scope and Literature-Selection Approach

This article was developed as a narrative review of the literature on multimodal plant–animal communication. Relevant studies were identified through searches of Scopus, Web of Science, PubMed, Google Scholar, ScienceDirect, and journal platforms of the University of Anbar. No formal lower publication-date limit was imposed, allowing inclusion of older foundational studies when directly relevant; the final literature search update was conducted in August 2026. Search terms included combinations of “plant–animal communication”, “multimodal signaling”, “plant volatiles”, “pollinator communication”, “plant bioacoustics”, “herbivore-induced signaling”, “sensory ecology”, and related terms reflecting the major themes of the review. No formal language restriction was applied; however, English-language, peer-reviewed publications directly relevant to plant–animal information exchange were prioritized, with particular emphasis on primary research articles that linked signal or cue production to receiver detection, behavioral or physiological responses, and ecological outcomes. Primary research articles were preferentially used to support specific mechanistic, behavioral, and ecological claims, whereas review articles were used primarily to provide broader conceptual context and to synthesize established patterns across the literature. Studies were excluded when their relevance to plant–animal communication was indirect, when they did not provide evidence connecting a plant- or animal-derived trait to receiver response, or when they fell outside the thematic scope of the review. Because this was a narrative rather than a systematic review, a formal prospective screening log was not maintained. A total of 117 publications were retained in the final reference set. Additional records were also considered during literature selection but were not retained when they did not sufficiently meet the thematic and relevance criteria. Representative studies included in Table 1 were selected to illustrate major communication modalities and to provide examples spanning signal or cue production, receiver detection, behavioral or physiological response, and ecological consequence. The strength of evidence was assessed qualitatively rather than through a formal meta-analytic grading procedure. Throughout the review, candidate communication traits and mechanisms were evaluated using a common evidence chain comprising: (i) production or modification of the putative signal or cue; (ii) detection by an ecologically relevant receiver; (iii) a reproducible behavioral or physiological response; (iv) measurable plant-level benefits, costs, or other ecological consequences; (v) evidence for adaptation, receiver exploitation, or reciprocal selection, where relevant; and (vi) validation under natural or ecologically realistic conditions. Evidence was considered stronger when multiple links in this chain were demonstrated experimentally and when comparable findings were supported across multiple studies or biological systems. Evidence was considered more limited when support was restricted to trait production, physiological response, receiver detection, or ecological association without establishing the broader communication pathway. These criteria form the basis of the evidence-strength categories summarized in Table 2. Because the literature was selected and synthesized using a qualitative narrative approach rather than a systematic-review protocol, the evidence base should not be considered exhaustive and may be subject to selection bias despite the use of multiple literature sources and predefined thematic criteria.

## 2. Multimodal Signaling in Plant–Animal Communication

Plant–animal interactions often involve multiple sensory traits, but the simultaneous presence of several traits does not necessarily demonstrate multimodal signaling. A multimodal interaction is established more convincingly when an animal receiver detects and integrates information from two or more sensory channels during decision-making. Depending on their functional relationship, these components may act as redundant signals that convey similar information, complementary signals that provide different but jointly useful information, independent cues that influence behavior without integration, synergistic signals whose combined effect exceeds their separate effects, or sequential signals used at different stages of resource localization and evaluation [1,2,11]. Distinguishing among these alternatives is important because the ecological meaning of a multimodal display depends on how the receiver actually processes and uses the available information. Chemical signals are among the most extensively studied components of plant–animal communication. Plant volatile organic compounds (VOCs) can attract pollinators, guide herbivores to suitable hosts, recruit natural enemies, and provide information about plant identity or physiological condition [2]. Fruit scent can also function as an evolved cue facilitating seed dispersal by animal receivers [4]. Detailed aspects of VOC blend composition, transmission, and receiver responses are addressed in Section Plant Volatile Information in Host Location, Foraging, and Indirect Defense. Visual signaling is likewise central to many plant–animal interactions. Floral color, shape, contrast, and patterning can guide pollinators toward nectar and pollen rewards, whereas fruit coloration can help dispersers identify ripe fruits [3,4]. In some systems, visual traits are thought to evolve in close association with the sensory capacities of animal receivers, thereby increasing the efficiency of attraction and recognition [1]. Directional visual cues may also reduce search time and enhance the probability that an animal reaches the relevant reproductive or nutritional structure.

Gustatory information becomes particularly important after direct contact with a floral reward or plant tissue, when animals can assess chemical properties that are not necessarily evident from visual or olfactory cues alone. In floral nectar, gustatory evaluation includes both sugar concentration and sugar identity; honeybees can discriminate among naturally occurring nectar sugars and show different preferences for sucrose, glucose, maltose, and fructose [12]. Nectar is also chemically more complex than a simple carbohydrate reward. Secondary metabolites, including alkaloids and other non-sugar constituents, can occur together with sugars and modify the attractiveness, palatability, and consumption of floral rewards. In *Nicotiana attenuata*, for example, nectar secondary metabolites, including nicotine, altered nectar removal and the behavior of moth, hummingbird, and ant visitors [13].

Non-sugar nectar compounds may produce effects ranging from deterrence to modification of learning and reward perception. Caffeine naturally occurring in the nectar of *Coffea* and *Citrus* enhanced honeybee memory of a learned floral odor at concentrations below the bees’ bitter-taste threshold, demonstrating that nectar chemistry can influence subsequent foraging behavior as well as immediate reward evaluation [14]. Conversely, animals can use gustatory and postingestive information to avoid potentially harmful resources. Honeybees can learn to avoid odors associated either with aversive taste or with the adverse postingestive consequences of toxic compounds, showing how contact-based chemical assessment can influence later behavioral decisions [15]. Gustatory information therefore functions as an important short-range component of multimodal decision-making, linking initial attraction to resource acceptance, avoidance, and experience-dependent modification of subsequent visits.

Floral electric fields represent an emerging and comparatively specialized component of plant–animal information transfer. Bumblebees can detect experimentally measured floral electric fields and learn to use them during flower discrimination [16]. However, the current evidence base is concentrated in a limited number of pollinator systems, and the consequences for plant reproductive success, populations, and communities remain insufficiently quantified. Electrical cues are therefore treated here as supplementary or emerging sensory information rather than as a broadly established communication modality. Thus, current evidence establishes the physical presence of floral electric fields, their detection by bumblebees, and learned use in behavioral discrimination, but does not yet establish broad ecological consequences under natural conditions or demonstrate that these fields evolved specifically for communication.

Acoustic and vibrational cues remain less well understood, but they represent an emerging area of plant–animal communication research. In particular, evidence from bat-pollinated and bat-dispersed plants suggests that some plant structures can modify or enhance reflected acoustic signals, thereby improving their detectability to echolocating animals [17]. This does not necessarily mean that all plants actively produce sound as intentional signals; however, it does indicate that acoustic properties can contribute to ecological communication in certain systems.

Taken together, these sensory channels may operate independently or, in systems where receiver integration is demonstrated, contribute to multimodal signaling. Their combined effects may be additive, synergistic, complementary, or context dependent, depending on how the receiver detects and uses the available information. A pollinator, for example, may use floral color at long range, scent at intermediate range, and gustatory or electrical information at close range. In the same way, herbivores and seed dispersers may combine visual, chemical, and contact-based cues when evaluating plant resources. Multimodal integration can therefore improve signal reliability, reduce uncertainty, and enhance communication efficiency under ecologically variable conditions [2,11].

The main signaling modalities involved in plant–animal communication and their possible functional roles are summarized in Figure 1. This conceptual overview highlights how different sensory channels may act independently or in combination to shape ecological outcomes such as pollination, seed dispersal, host location, and herbivore deterrence.

### Plant Volatile Information in Host Location, Foraging, and Indirect Defense

Plant volatile organic compounds (VOCs) constitute a chemically diverse source of information in plant–animal interactions, but their ecological functions depend strongly on the conditions under which they are produced and perceived. Plant volatile emissions can be broadly distinguished between constitutive or background emissions and induced emissions. Constitutive bouquets may vary among plant species, genotypes, developmental stages, and times of day, thereby providing animals with information potentially associated with plant identity, physiological state, or resource availability [2,18]. By contrast, herbivore-induced plant volatiles (HIPVs) are released or quantitatively modified following herbivore attack and can differ substantially from constitutive emissions [19,20]. These categories are not mutually exclusive, because herbivory may alter the quantity or relative abundance of compounds that are already present in the constitutive bouquet rather than generating an entirely novel chemical profile.

Source attribution is therefore essential when interpreting plant-associated volatiles. Floral attractants released from reproductive tissues should be distinguished from constitutive vegetative emissions, herbivore-induced plant volatiles, and broader stress-associated emissions. Volatiles detected following herbivory or tissue damage may reflect regulated plant induction, direct release from damaged tissues, or other components of the surrounding odor environment. Potential microbial contributions, including fungal volatiles, should likewise be considered when the biological source of an emitted compound is uncertain; such non-plant chemical sources are considered further in Section 8.

The biological activity of plant odors also cannot be inferred from the presence of individual compounds alone. Animal receivers frequently respond to combinations of compounds whose identity, concentration, relative proportion, and release rate determine the information conveyed. In the grapevine moth *Lobesia botrana*, for example, host finding was elicited by a ratio-specific blend of three common plant volatiles rather than by plant-specific compounds, and females responded to the blend at release rates of only a few nanograms per minute [21]. This illustrates how common volatile constituents can acquire receiver-specific informational value through their quantitative relationships within a blend. During atmospheric transport, these relationships may change because compounds differ in reactivity and dispersion; consequently, alteration of blend composition or ratios can reduce successful host location even when many of the original compounds remain detectable [22,23].

Plant VOCs can serve different ecological functions depending on the receiver. Herbivores may exploit volatile bouquets to identify suitable host plants, feeding sites, or oviposition resources, whereas pollinators may use floral odors during resource location and discrimination [2,22,24]. Herbivore-induced emissions can also contribute to indirect plant defense by recruiting predators or parasitoids of herbivores. A classic experimental example showed that maize seedlings damaged by caterpillars and exposed to caterpillar oral secretions released induced terpenoid volatiles that were exploited by the parasitoid *Cotesia marginiventris* during host location [25]. Related work has demonstrated that constitutive and herbivore-induced volatile profiles can differentially influence the attraction of biological-control agents [19], and broader evidence supports an important role for HIPVs in multitrophic interactions [20]. Thus, the same volatile compound or blend may function as resource-location information for one receiver, indirect-defense information for another, or even be exploited by herbivores themselves.

Herbivore-induced plant volatiles should also be considered within a multi-receiver information network rather than as compounds directed toward a single trophic partner. HIPVs may be perceived by distant tissues of the emitting plant, neighboring plants, herbivores, and diverse carnivorous organisms, including predators and parasitoids. Importantly, the evolutionary interpretation of these emissions remains context dependent: some volatile compounds may function as evolved signals, whereas others may represent damage-associated cues or physiological consequences of herbivory that receivers have evolved to exploit. Evaluating HIPVs therefore requires identifying the relevant receiver, the resulting behavioral or physiological response, and the fitness consequences for the emitting plant under ecologically realistic conditions [26].

From a broader tritrophic perspective, herbivore-induced volatile emissions and extrafloral nectar secretion can function as complementary forms of indirect plant defense by recruiting or supporting natural enemies of herbivores. These traits may interact at physiological and ecological levels, illustrating that plant defensive signaling is embedded within multitrophic networks rather than operating through isolated plant–herbivore pairs [27].

Plant-derived volatile compounds can also induce avoidance rather than attraction. In behavioral assays with the peach fruit fly, *Bactrocera zonata*, methyl salicylate produced consistent repellence, whereas the responses to thymol and limonene differed according to compound identity and exposure time, further illustrating that insect behavioral responses cannot be inferred from the presence of a volatile compound alone [28].

Receiver responses to plant volatiles are also plastic rather than fixed. Experience and associative learning can alter the behavioral value assigned to an odor. In the parasitoid system described above, females learned to exploit plant-produced volatiles more effectively after experiencing them in association with hosts or host-related material [25]. Similarly, pollinators can learn associations between floral odors and rewards, modifying subsequent odor preference and flower choice [24]. Consequently, demonstrating olfactory communication requires more than showing that an animal can physiologically detect a compound; behavioral assays should establish whether detection changes orientation, attraction, avoidance, host choice, or other ecologically relevant decisions.

Volatile production may also involve physiological and ecological trade-offs. Biosynthesis and induced emission require metabolic investment, and experimental work in maize has shown associations between the intensity of induced volatile production and reductions in plant performance; however, because volatile induction occurs together with other defensive responses, such costs cannot always be attributed exclusively to VOC production [29]. The ecological balance of costs and benefits is likewise context dependent: induced emissions may benefit a plant when they recruit effective natural enemies, whereas the same chemical information may be exploited by herbivores or may provide little benefit when appropriate natural enemies are absent [20,29].

Accordingly, the detection of a volatile compound in plant headspace should not by itself be interpreted as evidence of a communication function. Depending on the available evidence, an emitted VOC may represent a metabolic by-product, an incidental cue exploited by a receiver, or an evolved communication signal; these categories should not be treated as interchangeable. Stronger evidence requires demonstrating the biological pattern of production or modification, exposure of the receiver at ecologically realistic concentrations and blend ratios, sensory detection, a reproducible behavioral or physiological response, and a relevant ecological consequence. Environmental conditions, background vegetation, atmospheric chemistry, receiver experience, and community composition can all modify these links [20,22,23]. Plant volatile communication should therefore be evaluated as a complete source–transmission–receiver–response pathway rather than inferred solely from chemical presence.

## 3. Animal-Derived Elicitors and Effectors in Plant Signaling

Herbivory can induce substantial changes in plant metabolism and signaling, including alterations in the production and composition of volatile organic compounds. The nature of these responses depends partly on the feeding strategy of the herbivore, because chewing insects and sap-feeding insects cause different patterns of tissue damage and expose plants to different sets of animal-derived cues [30]. These differences allow plants to distinguish, at least to some extent, between mechanical injury and biologically induced damage.

A growing body of evidence shows that plants can perceive molecules present in insect saliva, oral secretions, and other herbivore-associated materials. These compounds may function either as elicitors that activate plant defenses or as effectors that suppress or modify those responses [31]. Recognition of such cues enables plants to initiate signaling pathways that can influence local defense, systemic responses, and the production of herbivore-induced volatile compounds.

Salivary effectors provide a clear example of how herbivores can manipulate plant responses. In *Spodoptera frugiperda*, the salivary effector SfPDI has been shown to modulate plant defense responses in a manner that improves herbivore foraging efficiency [32]. Such findings demonstrate that animal-derived molecules are not merely by-products of feeding but can actively influence the physiological response of the plant.

Several herbivore-associated molecular patterns have also been identified as elicitors of plant defense. β-Glucosidase from the oral secretions of *Pieris brassicae* has been described as an elicitor capable of inducing plant defensive responses [33]. Another well-known example is *N*-(17-hydroxylinolenoyl)-*L*-glutamine, commonly known as volicitin, which was originally identified in the oral secretions of beet armyworm (*Spodoptera exigua*) larvae and is associated with the induction of plant volatile emissions [34].

Animal-derived compounds can also suppress plant defense. Glucose oxidase, a salivary enzyme produced by some caterpillars, can modify wound-induced responses and alter the expression of plant defenses following herbivore attack [35]. The coexistence of elicitors and suppressors within herbivore secretions illustrates the dynamic nature of plant–herbivore interactions, in which plants attempt to recognize and respond to attack while herbivores evolve mechanisms that reduce or redirect those responses.

Taken together, these interactions show that communication between plants and herbivores is bidirectional. Plants respond not only to physical damage but also to specific biochemical information associated with the feeding animal, while herbivores can actively modify plant signaling through compounds released during feeding. This reciprocal exchange of information forms an important component of plant defense and may generate selective pressures on both plants and herbivores; however, reciprocal coevolution should be inferred only when evolutionary change in both interacting partners is demonstrated.

## 4. Mutualism, Signal Reliability, and Ecological Outcomes in Plant–Animal Communication

Plant–animal communication encompasses interactions that range from mutualistic relationships to antagonistic or exploitative associations. In mutualistic systems, both partners may benefit from the exchange of information, as occurs when plants attract animals that provide services such as pollination, seed dispersal, or protection from herbivores. However, these benefits are rarely free of costs. For example, protective ants visiting flowers may reduce herbivore pressure while simultaneously imposing variable costs on pollination, illustrating how the ecological outcome of an interaction depends on the balance between benefits and costs experienced by the plant [36]. Communication should therefore be considered within the broader ecological context in which signals are produced, detected, and acted upon.

Extrafloral nectaries (EFNs) provide a particularly informative example of how plant traits can shape multitrophic interaction networks beyond simple pairwise mutualisms. By supplying carbohydrate-rich rewards, EFNs can recruit ants and other arthropods that contribute to indirect defense against herbivores. However, their ecological roles are multifunctional and strongly context dependent. In addition to enhancing plant protection, EFNs can alter the spatial distribution and activity of ants on plants, influence interactions with pollinators and trophobiotic insects, and contribute to the organization of broader arthropod food webs. Their net effects therefore depend on nectary location, plant phenology, ant identity and activity, herbivore pressure, pollination context, and the wider community of interacting organisms [37,38].

The ecological outcomes of EFN-mediated interactions may also depend on the simultaneous availability of other ant-associated food resources. In a tropical shrub, field experiments showed that the combined presence of extrafloral nectar and trophobiotic resources influenced ant activity and plant–insect interactions, with consequences for herbivory, pollinator activity, and plant performance. These findings demonstrate that the effects of ant–plant mutualisms cannot always be predicted from a single resource or pairwise interaction, because multiple resource pathways can jointly modify plant defense, pollination, and fitness within the same ecological network [39].

These examples also highlight the importance of interpreting plant–animal relationships as interaction networks rather than as isolated pairwise associations. Individual plants may simultaneously interact with herbivores, pollinators, ants, trophobiotic insects, predators, parasitoids, and other organisms, so changes in one interaction can indirectly modify several others. Resource provisioning through EFNs may therefore influence not only ant visitation and herbivore suppression but also pollinator activity, natural-enemy dynamics, and ultimately plant fitness. The direction and magnitude of these indirect effects depend on community composition, resource availability, and ecological context [37,39].

The reliability of a plant-derived signal is particularly important when animals use it to assess resource quality, host suitability, or the presence of competitors and natural enemies. Herbivore-induced plant volatiles can modify the behavior of insects and may influence both host-plant and mate-location processes. In moths, exposure to a herbivore-induced volatile has been shown to interfere with olfactory signaling pathways involved in host and mate location [40]. Such effects demonstrate that plant volatiles can influence animal behavior in ways that extend beyond simple attraction or repellence and may alter the ecological relationships among plants, herbivores, and other organisms. The relationship between signal emission, receiver perception, and major ecological outcomes is summarized conceptually in Figure 2.

Plant-mediated communication can also influence interactions involving omnivorous predators. Omnivores may feed on both plant and animal resources, and their net effect on plants can therefore vary according to feeding behavior and ecological context. Experimental evidence indicates that plant feeding by omnivorous predators can impose measurable costs on plant growth, even when these organisms also contribute to herbivore suppression [41]. At the same time, plant volatile emissions can attract omnivorous natural enemies. For example, constitutive and herbivore-induced systemic volatiles from *Salix* have been shown to differentially attract the omnivorous biocontrol agent *Anthocoris nemorum* [19]. These findings illustrate how plant signals can recruit animals whose ecological effects are beneficial under some conditions but costly under others.

Herbivory itself may also produce more complex outcomes than simple tissue loss. In potato plants, infestation by *Tecia solanivora* has been associated with increased tuber starch accumulation [42]. This finding suggests that plant responses to herbivory can involve substantial physiological and metabolic adjustments, although such responses should not automatically be interpreted as evidence that herbivory is beneficial to the plant. The ecological consequences of herbivore attack therefore depend on the intensity of damage, plant physiological responses, and the broader interactions among plants, herbivores, and their natural enemies.

More clearly mutualistic examples occur in specialized pollination systems in which plants provide food or breeding resources in exchange for pollination services. A documented brood-pollination mutualism occurs between *Stellera chamaejasme* and the flower thrips *Frankliniella intonsa*, demonstrating that insects with herbivorous or florivorous life-history traits may nevertheless provide direct reproductive benefits to plants under particular ecological conditions [43]. Such relationships highlight the difficulty of assigning fixed roles such as “beneficial” or “harmful” to animal partners, because the same interaction may involve both costs and benefits at different stages of the plant or animal life cycle.

Highly specialized plant–insect systems further illustrate how reciprocal adaptation can shape communication and reproductive dependence. Fig–wasp associations, for example, involve strong morphological and ecological constraints that influence access to reproductive structures and the success of both partners [44]. These specialized interactions demonstrate that successful plant–animal communication often depends not only on the presence of a signal but also on the ability of the receiver to interpret that information within a specific ecological and evolutionary context.

Overall, the ecological consequences of plant–animal signaling cannot be classified simply as mutualistic or antagonistic. Signal production, receiver response, resource availability, feeding strategy, and community context all influence whether an interaction ultimately benefits the plant, the animal, or both. Reliable signaling may facilitate pollination, seed dispersal, host recognition, or recruitment of natural enemies, whereas exploitation or conflicting interests can reduce these benefits. Plant–animal communication is therefore best understood as a dynamic ecological process in which signal reliability and behavioral responses are shaped by selection pressures and changing environmental conditions, which may become reciprocal in systems where selection by both interacting partners has been demonstrated.

## 5. Acoustic and Vibrational Cues in Plant–Animal Interactions

Acoustic and vibrational information represents a distinct but comparatively less explored component of plant–animal interactions. Acoustic signals propagate as pressure waves through fluid media such as air or water, whereas vibrational signals are transmitted through solid substrates. This distinction is important because the physical medium determines how information is generated, transmitted, and detected by a receiver [17]. Vibrational communication is particularly well developed in many insects, which use substrate-borne vibrations for functions such as mate localization, competition, and predator avoidance [45].

Plant tissues can themselves serve as mechanical transmission media for animal-generated vibrations. In many plant-dwelling insects, substrate-borne signals propagate through stems, petioles, leaves, and leaf veins, while the physical properties of different plant structures can filter signal frequency and amplitude and thereby influence signal transmission [46]. Experimental work on the harlequin bug *Murgantia histrionica* showed that vibratory courtship signals are transmitted through cabbage tissues, with particularly effective transmission along leaf veins, and that transmission characteristics may provide information useful for locating conspecifics on the plant [47]. More broadly, substrate-borne vibrations are used not only in mate localization and courtship but also in competitive, defensive, predator–prey, and other ecological interactions, and they can form part of multimodal communication networks [45]. Thus, plant structures can function as the physical channel through which vibrational information is transmitted between animal senders and receivers.

The effectiveness of acoustic information depends strongly on the physical environment through which it travels. Signal intensity generally decreases with distance, while transmission can also be influenced by frequency, background noise, and properties of the surrounding medium. Environmental noise may reduce signal detectability by masking biologically relevant sounds or decreasing the contrast between a signal and its acoustic background [48]. Consequently, the usefulness of acoustic information depends not only on sound production but also on whether the receiver can detect and discriminate that information under prevailing environmental conditions.

Acoustic interactions also involve ecological costs and constraints. Signals that are detectable by an intended receiver may simultaneously be detected by other organisms. In animal systems, predators can exploit acoustic information produced by prey, illustrating the potential cost of eavesdropping in communication networks [49]. Such interactions demonstrate that acoustic signaling, like chemical or visual signaling, is shaped by a balance between detectability and the risk of unintended interception.

As defined in the Introduction, physiological sound production, acoustic cueing, and functional signaling are treated as distinct evidential categories. Accordingly, plant-generated sounds are interpreted conservatively unless receiver detection and an ecologically relevant response are demonstrated [50]. Evidence for acoustic involvement in plant–animal interactions is currently stronger in systems where plant structures influence sounds generated by animals than in cases of demonstrated active acoustic signaling by plants. Bat–plant interactions provide an important example, because the morphology of some flowers and fruits can modify reflected ultrasonic signals and thereby affect their detectability to echolocating animals [17]. These systems show that plants can participate in acoustic interactions even when they are not themselves producing the initiating sound.

The following sections apply this evidential framework to plant sound production and perception and to the better-established echo-acoustic interactions between plants and animal partners.

## 6. Sound Production and Perception in Plants: Evidence and Limitations

The possibility that plants produce and respond to acoustic or vibrational stimuli has attracted increasing attention in recent years. Unlike animals, plants lack specialized auditory organs and a nervous system; nevertheless, they possess mechanosensitive cellular structures that can respond to mechanical forces and vibrations. Current research therefore approaches plant acoustics primarily through the mechanisms of sound production, mechanical perception, and physiological response rather than through assumptions of hearing in the animal sense [8,51].

Plants can generate detectable sounds as a consequence of physiological and mechanical processes, including changes in xylem tension, cavitation-related events, tissue movement, and processes associated with water transport and cellular activity [8,52]. These emissions establish sound production but, under the evidential framework defined above, do not by themselves establish a communication function [50]. Evidence that plants can respond to externally applied sound or vibration has also stimulated interest in potential acoustic perception. Experimental studies have reported physiological and developmental responses to mechanical or acoustic stimulation, although the underlying mechanisms remain incompletely resolved [8,50]. Mechanosensitive ion channels provide one plausible route through which physical vibrations could be converted into cellular signals, because these channels respond to changes in membrane tension and other mechanical forces [51]. However, demonstrating a physiological response to vibration is not equivalent to demonstrating acoustic communication.

This distinction is particularly important when interpreting plant-to-plant interactions. Experiments designed to exclude conventional cues such as direct physical contact, light, and some chemical signals have been interpreted as possible evidence that plants can detect information associated with neighboring individuals [53]. These findings are intriguing, but they do not yet establish that sound is the sole information carrier or that the observed response represents an evolved auditory communication system. Alternative mechanical or environmental pathways must be excluded before such conclusions can be considered definitive.

Applying the evidential framework defined above, a claim of acoustic communication requires a reproducible sound produced in a defined biological context, detection by an appropriate receiver, a consistent behavioral or physiological response, and an ecologically meaningful consequence [50,52]. At present, mechanosensitive signaling and plant-generated sounds support the plausibility of acoustic responsiveness, but evidence for ecologically functional plant–animal acoustic communication remains limited [8,51,52]. Stronger experimental evidence will be required to determine when these sounds function merely as physiological by-products, when they provide usable ecological cues, and when they meet the more stringent criteria for biological communication. Accordingly, where receiver detection, reproducible receiver responses, ecologically realistic consequences, or evidence of evolutionary specialization have not been demonstrated, proposed communicative roles of plant-generated acoustic or vibrational phenomena are treated here as hypotheses rather than as established communication systems.

## 7. Echo-Acoustic Interactions in Bat–Plant Mutualisms

Among the different forms of acoustic interaction between plants and animals, bat–plant mutualisms provide some of the clearest evidence that plant structures can influence the acoustic information available to animal receivers. Echolocating bats emit ultrasonic calls and evaluate returning echoes to detect and identify objects in their surroundings. In densely vegetated environments, however, echoes from leaves, stems, and other background structures can make floral or fruit targets difficult to distinguish. Plant traits that increase acoustic contrast may therefore improve detection by bats and enhance the efficiency of pollination or seed dispersal [17].

Bat-pollinated plants provide particularly informative examples of this process. Comparative analyses have shown that flowers associated with bat pollination can possess distinctive acoustic properties that differ from those of flowers adapted to other pollination systems [54]. These properties are largely determined by floral morphology and the way plant surfaces reflect incoming ultrasonic calls. Rather than producing the initiating sound themselves, such plants modify the echoes generated by an approaching bat, creating a form of passive acoustic signaling or acoustic cueing.

In some bat-pollinated cacti, specialized inflorescence structures can further increase the contrast between the flower and its acoustic background. An ultrasound-absorbing zone surrounding the floral structure has been shown to reduce interfering echoes and enhance the echo-acoustic contrast of the flower [55]. This mechanism is particularly relevant in cluttered vegetation, where target detection depends on the ability of the receiver to discriminate biologically important echoes from surrounding acoustic noise.

Acoustic traits may also contribute to fruit detection and seed dispersal. Some bat-dispersed plants display fruits away from dense foliage, thereby increasing their accessibility and acoustic visibility to flying bats. Studies of flagellichorous cucurbits have shown that bats can combine echolocation with olfactory information when locating ripe fruits [56]. Such systems demonstrate that acoustic information often operates together with other sensory modalities rather than functioning in isolation.

These bat–plant systems represent passive echo-acoustic interactions: bats generate the ultrasonic signal, while plant morphology modifies the returning echo [17,54,55]. Such interactions can influence pollination and seed dispersal without requiring active sound production by the plant.

By contrast, evidence that plant-generated sounds are consistently used by animal receivers remains limited; therefore, the evidential criteria defined above should be applied before inferring an active signaling function [17]. Overall, bat–plant systems demonstrate how plant morphology can become integrated into the sensory world of animal partners. By modifying echo intensity, spectral structure, or acoustic contrast, flowers and fruits may become easier for echolocating animals to detect. These interactions represent a specialized form of multimodal plant–animal communication in which acoustic information can operate alongside olfactory and other sensory cues to improve resource localization and mutualistic exchange.

## 8. Semiochemicals at the Plant–Animal Interface

Chemical information at the plant–animal interface includes plant-derived volatile organic compounds (VOCs), animal-produced chemical signals, and other semiochemicals that can influence the behavior or physiology of intended or unintended receivers [5,57]. The term semiochemical encompasses chemical cues and signals used in biological interactions, and their ecological function depends on the identity of the emitter and receiver and on the consequences of the interaction [5,6]. In interspecific interactions, such compounds may function as allomones, kairomones, or synomones according to the distribution of benefits and costs between the interacting organisms [5,6].

Animal pheromones are primarily involved in communication among conspecifics [57,58], but they become directly relevant to plant–animal communication when pheromone-mediated behavior occurs within a chemically complex environment shaped by plants. Host-plant volatiles can enhance, suppress, or otherwise modify insect responses to sex pheromones, and some phytophagous insects can acquire plant-derived compounds that subsequently contribute to pheromone production or act as pheromone precursors [59]. Thus, plant VOCs and animal pheromones should not always be considered as independent signaling systems; rather, they may interact within the same olfactory environment and jointly influence host location, mate finding, and behavioral decision-making.

Chemical signals may also be intercepted by organisms other than their intended receivers. Bark beetle pheromones, for example, occur within a semiochemical landscape that includes host-tree and fungal volatiles, creating opportunities for several species to respond to overlapping sources of chemical information [60]. Predators and parasitoids can exploit pheromones and other prey- or host-associated chemical cues as kairomonal information during foraging [61]. This form of ecological eavesdropping links animal chemical signaling to plant-associated multitrophic interactions and can contribute to the recruitment and foraging efficiency of natural enemies in biological-control systems [61].

These relationships are summarized in Figure 3, which emphasizes the shared chemical environment at the plant–animal interface. Plant-derived VOCs and animal-derived semiochemicals may be detected by pollinators, herbivores, predators, parasitoids, and other receivers, and their combined effects can alter behavior and ecological outcomes. Within this framework, allomones benefit the emitter while disadvantaging the receiver, kairomones benefit the receiver while being neutral or detrimental to the emitter, and synomones benefit both interacting organisms [5,6]. Importantly, the informational meaning of a chemical compound or blend is context dependent; the same chemical information may have different ecological consequences for different receivers [5,61].

Accordingly, the relevance of semiochemicals to plant–animal communication lies not in animal pheromone biology in isolation, but in the intersection among plant-derived chemicals, animal-produced signals, receiver sensory systems, and ecological context. This interface provides an important mechanistic connection between plant chemistry, animal behavior, ecological eavesdropping, and multitrophic interactions and leads directly to the sensory mechanisms of chemical detection discussed in Section 9.

## 9. Olfactory Detection of Plant Volatiles and Pheromones: Receptor Mechanisms and Evolution

Olfaction is a major sensory pathway through which animals obtain chemical information from their environment, including plant-derived volatile organic compounds (VOCs), pheromones, and other semiochemicals. Although vertebrates and insects employ molecularly distinct olfactory receptor systems, both rely on specialized sensory neurons and neural processing pathways that enable the detection and discrimination of biologically relevant odorants [62,63]. Rather than operating through a simple one-compound–one-receptor mechanism, olfactory perception frequently involves patterns of receptor activation that allow animals to distinguish complex odor mixtures [62].

In plant–insect interactions, olfactory perception is particularly important for host location, feeding, oviposition, and avoidance of unsuitable or damaged plants. Phytophagous insects detect plant VOCs primarily through olfactory sensilla located on the antennae, where odorant molecules interact with components of the peripheral olfactory system and activate olfactory sensory neurons [64]. Host recognition often depends on the composition and relative proportions of compounds within a volatile blend rather than on the presence of a single compound alone [59,64]. Consequently, plant odors can provide insects with information about plant identity, physiological condition, and suitability as feeding or oviposition sites.

Pheromone detection represents a specialized component of chemical perception. In moths, for example, female-produced sex pheromones are detected by pheromone receptors expressed in olfactory sensory neurons within antennal sensilla [65]. These receptors differ in ligand selectivity; some are narrowly tuned to particular pheromone components, whereas others respond to a broader range of structurally related compounds [65]. Such receptor specialization contributes to the recognition of species-specific pheromone blends and is therefore important for mate location and reproductive isolation.

Olfactory systems are also subject to evolutionary diversification. Changes in receptor gene repertoires, receptor expression, and ligand sensitivity can alter an animal’s ability to detect ecologically important chemicals [63,65]. Selection acting on both chemical signal production and receiver sensitivity can consequently promote increasingly specialized associations between signals and their receptors. However, the evolution of a pheromone cannot be explained solely by the ability of an animal to detect a chemical compound. For a chemical to function as a pheromonal signal, its production and perception must become consistently associated with a behavioral or physiological response that has biological significance within the species [57,62].

Importantly, pheromone perception does not occur independently of the surrounding chemical environment. Host-plant volatiles can enhance, suppress, or otherwise modify insect responses to sex pheromones, and some phytophagous insects can acquire plant-derived compounds that subsequently contribute to pheromone production or act as pheromone precursors [59]. Such interactions demonstrate that plant-derived signals and intraspecific chemical communication can operate simultaneously, allowing insects to integrate information about potential mates with information about host plants and habitat quality.

In mammals, social chemical information can be processed through the main olfactory system, the vomeronasal system, or both, depending on the species and the chemical involved [66]. The vomeronasal organ is particularly important in many mammals for detecting pheromones and other socially relevant chemical cues, with information subsequently transmitted through the accessory olfactory bulb to brain regions associated with reproductive, social, and neuroendocrine responses [66]. Nevertheless, substantial variation occurs among mammalian lineages, and the relative roles of the main olfactory and vomeronasal systems should not be generalized across vertebrates [66].

Overall, olfactory communication emerges from interactions among chemical composition, receptor sensitivity, neural processing, and ecological context. The evolutionary diversification of these components allows animals to distinguish plant VOCs, pheromones, and other semiochemicals within complex natural odor environments. This sensory flexibility provides an important mechanistic link between plant signaling and animal behavior, influencing host selection, mate recognition, foraging, and the evolution of plant–animal interactions.

## 10. Leaf Coloration as a Visual Signal in Plant–Animal Interactions

Leaf coloration is primarily determined by photosynthetic and accessory pigments, but variation in leaf color can also influence how animals perceive and interact with plants. Although green coloration predominates because of chlorophyll, leaves may also display red, purple, yellow, bronze, or variegated patterns as a result of differences in pigment composition, developmental stage, or environmental conditions. Seasonal color changes during senescence can involve declining chlorophyll concentrations together with increasing anthocyanin accumulation, as demonstrated in Japanese maple leaves [67]. Red or purple coloration may also occur specifically on the lower leaf surface, a trait that has evolved repeatedly across plant lineages and may have several physiological and ecological functions [68].

Importantly, conspicuous leaf coloration should not automatically be interpreted as a communication signal. Anthocyanins and other pigments can contribute to photoprotection, antioxidant activity, and tolerance to environmental stress, providing direct physiological benefits to the plant [69]. Geographic patterns in red and yellow autumn coloration have likewise been linked to variation in temperature and solar radiation, supporting physiological explanations for at least part of the observed variation in leaf color [70]. Thus, evidence that a leaf color is conspicuous is not sufficient to demonstrate signaling; a communication hypothesis requires evidence that animals can perceive the coloration, alter their behavior in response to it, and thereby generate a selective consequence for the plant.

Young red leaves provide a useful example of this distinction. Red coloration is common during early leaf development in many tropical and subtropical species and has frequently been associated with defense against herbivores. A comparative study involving 250 tropical plant species reported that young red leaves contained higher concentrations of anthocyanins and tannins and experienced less herbivore damage than young green leaves, suggesting a potential defensive function [71]. However, this does not necessarily mean that redness acts as a visual warning signal. In another comparative study, common lepidopteran and coleopteran herbivores were predicted to have limited ability to discriminate red from green young leaves, suggesting that anthocyanins may contribute to defense through physiological or chemical mechanisms rather than through visual signaling alone [72]. These contrasting findings illustrate why animal sensory capabilities must be considered when evaluating putative plant visual signals.

Autumn coloration has generated an especially active debate regarding plant–herbivore communication. The coevolutionary signaling hypothesis proposes that conspicuous autumn colors may provide herbivorous insects with information about the defensive condition of potential host trees. Under this interpretation, strongly colored trees may signal greater defensive investment and thereby reduce colonization by insects [73]. Nevertheless, this hypothesis remains debated, and physiological and anti-herbivory explanations are not necessarily mutually exclusive. A recent synthesis emphasized that red and yellow autumn coloration may arise from several interacting physiological and ecological functions and cautioned against treating any single explanation as universally applicable [74].

Overall, leaf coloration can influence plant–animal interactions, but its ecological meaning is strongly context dependent. In some systems, coloration may alter herbivore host choice or function as part of a defensive strategy, whereas in others it may primarily reflect physiological processes such as photoprotection, pigment turnover, or senescence. Demonstrating a true visual communication function therefore requires evidence linking leaf color to animal perception, behavioral modification, and measurable fitness consequences. This distinction is essential for separating visually conspicuous plant traits from traits that have evolved specifically as signals to animal receivers.

## 11. Herbivore Recognition and Induced Chemical Defense: From HAMP Perception to Specialized Metabolites

Plants possess sophisticated innate immune systems that allow them to distinguish mechanical injury from damage associated with feeding herbivores. Herbivore attack generates a combination of wound-derived signals and herbivore-associated molecular patterns (HAMPs), including molecules present in oral secretions, saliva, regurgitant, and other herbivore-derived materials. Recognition of these cues enables plants to activate defense responses that are more specific than those induced by mechanical damage alone [75]. However, the molecular receptors responsible for perceiving many known HAMPs remain unidentified, indicating that plant recognition of herbivores is still only partly understood [75].

Some HAMPs are perceived through plasma membrane-localized pattern-recognition receptors (PRRs), which initiate signaling processes analogous to pattern-triggered immunity. A well-characterized example is the inceptin receptor (INR), a leucine-rich repeat receptor-like protein that recognizes caterpillar-derived inceptin peptides in certain legumes and triggers defense responses that increase resistance to herbivory [76]. More recently, a conserved myosin-like protein from the salivary sheath of the small brown planthopper *Laodelphax striatellus* was shown to function as a HAMP and to induce BAK1-dependent immune responses in several plant species [77]. These findings provide direct evidence that plants can recognize specific animal-derived molecular signatures rather than responding solely to tissue damage.

Following herbivore perception, plants activate interconnected early signaling events that may include changes in cytosolic Ca^2+^ concentrations, mitogen-activated protein kinase (MAPK) activation, reactive oxygen species (ROS) production, membrane-associated signaling, and extensive phytohormonal reprogramming [75,77]. These responses can be initiated not only by mechanical tissue damage but also by herbivore-derived cues delivered in oral secretions, saliva, regurgitant, and oviposition-associated fluids. Jasmonates (JA) play a central role in many defenses against chewing herbivores, whereas salicylic acid (SA), ethylene, and abscisic acid (ABA) can modify the magnitude, timing, and specificity of the response through interacting signaling networks [75,78,79,80]. Egg- or oviposition-derived cues can also alter subsequent herbivore responses; for example, insect egg extracts can activate SA signaling and suppress JA-dependent defenses in *Arabidopsis*, thereby affecting subsequent caterpillar performance [79]. ABA has likewise been implicated in the activation of systemic tissues primed for JA-dependent herbivore resistance [80]. These hormonal pathways therefore operate as an interacting regulatory network rather than as isolated defense modules. 

Herbivores, in turn, can interfere with plant recognition and signaling by delivering effectors that suppress or redirect defense pathways [75]. Plant–herbivore interactions may generate opposing or reciprocal selective pressures on signal production and perception, although demonstrated reciprocal coevolution requires evidence of evolutionary responses in both partners. Evidence from the inceptin system illustrates this evolutionary dynamic: comparative analyses across legumes have revealed evolutionary gain and loss of functional HAMP recognition associated with diversification of the inceptin receptor locus [81]. Such variation demonstrates that the ability to recognize herbivore-derived molecules is itself an evolvable trait and can differ substantially among related plant lineages.

Defense signaling is not restricted to the site of herbivore attack. Local damage can initiate rapid long-distance signals that prepare undamaged tissues for subsequent stress. In *Arabidopsis*, wound-induced glutamate activates GLUTAMATE RECEPTOR-LIKE channels and generates propagating Ca^2+^ signals that transmit information from damaged leaves to distant tissues, where defense responses are subsequently activated [82]. This process is more accurately described as systemic wound or herbivory signaling rather than systemic acquired resistance, because the latter has a more specific meaning in plant immunity.

### Herbivore-Induced Specialized Metabolites: Direct and Indirect Defense

Plant chemical defense includes both constitutive and inducible components. Constitutive defenses are present before herbivore attack and can provide an immediate chemical barrier, whereas inducible defenses are synthesized, activated, or quantitatively enhanced after plants perceive feeding damage or herbivore-derived elicitors. These responses include a diverse range of specialized metabolites, including phenolic compounds and flavonoids, alkaloids, terpenoids, glucosinolates, benzoxazinoids, and other compounds that can alter herbivore feeding, digestion, development, survival, or host choice. Importantly, constitutive and inducible defense are not mutually exclusive categories: the same metabolite may be present at basal levels and subsequently increase after attack, while stored precursors can also be rapidly converted into biologically active products following tissue damage.

Phenolics and flavonoids provide examples of specialized metabolites associated with direct anti-herbivore defense. In maize, the C-glycosyl flavones maysin and apimaysin in silks have been genetically linked to antibiosis against the corn earworm *Helicoverpa zea*, with variation in these metabolites contributing significantly to variation in larval growth [83]. Alkaloids can likewise exert strong direct effects. In *Nicotiana attenuata*, experimental silencing of nicotine biosynthesis reduced constitutive and inducible nicotine by more than 95%; nicotine-deficient plants were preferred by some herbivores and experienced substantially greater leaf damage under natural field conditions, providing direct evidence for the defensive function of this alkaloid [84]. Other specialized compounds can produce similarly receiver-specific effects. In *Arabidopsis*, plants lacking aliphatic glucosinolates supported substantially greater larval growth of the generalist herbivore *Mamestra brassicae*, demonstrating a direct connection between metabolite production and herbivore performance [85].

Plant-derived essential oils can likewise contribute to direct anti-herbivore effects. Under greenhouse conditions, nanoemulsion formulations of thyme, clove, and lavender essential oils were evaluated against the cotton aphid, *Aphis gossypii*, on eggplant, providing an applied example of how plant-derived secondary compounds can contribute to direct pest suppression [86].

Terpenoids and other herbivore-induced plant volatiles provide an important bridge between chemical defense and plant–animal information exchange. Unlike nonvolatile compounds that act directly after herbivores contact or consume plant tissue, induced volatile blends can operate indirectly by modifying the behavior of organisms at higher trophic levels. In maize, caterpillar feeding together with caterpillar oral secretions induces the release of terpenoid volatiles that are exploited by the parasitoid *Cotesia marginiventris* during host location [25]. Jasmonate signaling provides a mechanistic link between direct and indirect defense: jasmonate-deficient tomato plants supported greater caterpillar survival and failed to show the normal herbivore-induced attraction of predatory mites, whereas exogenous jasmonic acid restored both defense components [78]. These experiments demonstrate that hormonal regulation can coordinate compounds that directly reduce herbivore performance with volatile information that recruits natural enemies.

Specialized-metabolite responses should nevertheless not be interpreted as uniformly beneficial to plants or as communication signals simply because their concentrations change after herbivory. Direct defensive metabolites such as nicotine, flavonoids, or glucosinolates may primarily function through toxicity, deterrence, or reduced digestibility, whereas volatile terpenoids can become informational cues for herbivores, predators, or parasitoids. Moreover, specialized herbivores may tolerate, detoxify, or even exploit compounds that deter generalists. The ecological interpretation therefore requires evidence linking metabolite production or induction to measurable outcomes such as herbivore feeding or performance, natural-enemy recruitment, plant damage, or plant fitness. This distinction connects herbivore-induced specialized metabolism to the broader evidence framework used throughout this review and avoids equating chemical production with communication per se.

These mechanisms have important implications for crop protection. Identification of HAMP receptors and conserved herbivore-derived elicitors may provide opportunities to enhance crop recognition of insect attack or introduce recognition capacities into plants that naturally lack them [76]. Likewise, manipulating herbivore-induced volatile responses or volatile-mediated priming may contribute to ecological pest management, although such approaches must account for genotype, environment, herbivore community, and the behavior of natural enemies [20,87]. Recent field-oriented work on volatile-mediated interactions among crop cultivars further indicates that specific combinations of responsive genotypes can reduce aphid pressure without necessarily compromising crop performance [87].

Overall, plant perception of herbivore-derived signals is best understood as an integrated component of innate immunity rather than as a simple defensive reflex. HAMP recognition, receptor evolution, systemic signaling, hormonal regulation, and indirect defense collectively connect molecular perception with herbivore behavior and multitrophic ecological interactions. A clearer understanding of these mechanisms may therefore contribute both to fundamental models of evolutionary interactions between plants and herbivores and to the development of more targeted and sustainable approaches to crop protection.

## 12. Plant Signals and Animal Perception: Sensory Integration, Learning, and Behavioral Responses

### 12.1. Sensory Ecology and Associative Learning

Animals encounter plant-derived information through sensory systems shaped by their ecological requirements and evolutionary histories. In plant–pollinator interactions, visual and olfactory traits are particularly important because they enable animals to locate flowers, discriminate among plant species, and associate floral characteristics with rewards [88]. Hymenopteran pollinators can perceive ultraviolet components of floral reflectance that are not visible to humans, demonstrating that floral appearance must be evaluated from the sensory perspective of the receiver rather than from human vision alone [89].

Detection or discrimination of a plant trait should not be equated with preference. Perception indicates that an animal can detect a stimulus, whereas preference requires demonstrated differential choice among alternatives; neither alone establishes a reproductive or fitness benefit to the plant.

Animal responses to plant signals are also strongly influenced by learning. Bees and other insect pollinators can form associations between floral colors, odors, and the quality or presence of nectar and pollen rewards, and these learned associations subsequently influence flower choice [24,88]. Floral scent is especially important in this context because pollinators can learn and remember odor–reward associations, potentially generating selection for distinctive and sufficiently reliable floral signals [24]. Thus, the ecological effect of a plant trait depends not only on its production by the plant but also on the sensory capabilities, previous experience, and behavioral responses of the animal receiver.

Learning is therefore relevant across multiple receiver guilds, although its evidence base is uneven: it is particularly well demonstrated in pollinators and parasitoids, whereas the extent to which experience modifies plant-cue use in herbivores and seed dispersers remains more system dependent and less consistently quantified.

### 12.2. Multimodal Signal Integration and Decision-Making

Plant-associated decisions may involve more than one sensory dimension, but the simultaneous presence of several traits does not by itself demonstrate multimodal integration. Evidence for multimodal signaling is stronger when experiments manipulate individual sensory components independently and in combination and then quantify the receiver’s response. Factorial designs can, for example, compare visual information alone, olfactory information alone, and combined visual–olfactory treatments, while analogous approaches can be used for reward-related, tactile, structural, electrical, or other sensory components. Such experiments allow investigators to determine whether combined effects are additive, synergistic or configural, redundant or backup, antagonistic or conflicting, context dependent, or functionally independent.

Experiments with the hawkmoth *Manduca sexta* provide an example of this approach. Visual and olfactory floral information can jointly influence foraging decisions, while previous experience modifies how moths subsequently weight these sensory components [11]. A complementary example is provided by the oligolectic bee *Hoplitis adunca*, in which behavioral experiments separating visual and olfactory floral cues showed that the blue coloration of *Echium vulgare* flowers primarily contributed to attraction, whereas host-specific olfactory cues were important for host recognition and discrimination from non-host plants [90]. This functional division between sensory channels illustrates how different modalities can provide complementary information rather than simply producing redundant attraction. Multimodal components may therefore serve different functional roles: redundant signals can provide overlapping information, complementary signals can contribute different but jointly useful information, and sequential signals can operate at different stages of resource localization and evaluation. By contrast, multiple traits that affect behavior independently should not automatically be classified as a single integrated multimodal signal. These empirical examples illustrate why multimodal communication should be inferred from experiments that isolate, recombine, or otherwise manipulate sensory components, rather than from the mere co-occurrence of several plant traits.

The interpretation of multimodal effects also requires consideration of receiver experience and ecological context. Flower-visiting insects may evaluate floral color, scent, morphology, nectar volume, and nectar concentration simultaneously, but the relative importance of these traits can change with learning, resource availability, and environmental conditions [88,91]. Accordingly, demonstrating multimodal communication requires evidence not only that several traits are present, but also that the receiver detects, combines, or sequentially uses them in a way that changes behavioral or physiological decision-making.

Receiver responses should therefore be interpreted within the ecological setting in which signals are encountered, including background vegetation, temperature, wind, humidity, time of day, predator presence, resource availability, and community composition, all of which can alter signal transmission, detectability, or behavioral value.

Rigorous experiments that manipulate multiple sensory modalities independently and factorially remain comparatively uncommon outside well-studied floral systems. Consequently, for many plant–animal interactions, the extent to which multimodal effects are additive, synergistic or configural, redundant, antagonistic, or strongly context dependent remains an important evidence gap.

### 12.3. Deception and Exploitation of Receiver Biases

Plant signals do not always indicate the presence of a corresponding reward. Deceptive pollination systems provide some of the clearest examples of plants exploiting pre-existing sensory preferences of animal receivers. Sexually deceptive orchids attract male insects by mimicking components of female mating signals, particularly sex-pheromone chemistry, often without providing a nutritional reward [92]. In these systems, pollinator attraction may also involve visual, morphological, or tactile components, although their relative importance differs among orchid–pollinator associations [92].

Deception does not imply that receivers remain behaviorally passive. Pollinators can learn to avoid unrewarding flowers or locations after unsuccessful visits, potentially imposing constraints on the frequency, spatial distribution, and phenotype of deceptive plants [92]. Consequently, deceptive signaling can generate a dynamic evolutionary relationship between signal production and receiver discrimination. The persistence of such systems depends on the balance between plant reproductive benefits, costs imposed on pollinators, receiver learning, and the ecological availability of alternative floral resources rather than on deception being universally advantageous.

### 12.4. Ecological, Evolutionary, and Applied Implications

The interaction between plant signals and animal sensory systems can generate reciprocal selective pressures. Animals that more effectively recognize rewarding, suitable, or harmful plants may gain fitness advantages, whereas plants can experience selection on traits that alter attraction, deterrence, or recognition by those animals [24,88]. These relationships are therefore dynamic rather than fixed, because changes in plant communities, animal sensory environments, or the temporal availability of resources can modify the information available to receivers.

Environmental change may disrupt these relationships even when the sensory mechanism itself remains intact. Global analyses indicate that climate change is producing unequal phenological shifts in plants and animals, increasing the potential for temporal mismatches between plant resources and the animals that depend on them [93]. Such divergence could reduce the effectiveness of otherwise well-established signaling and reward relationships if flowering, fruiting, herbivore activity, or pollinator emergence become increasingly asynchronous [93].

Understanding animal responses to plant traits also has practical relevance for agricultural ecosystem management. Flower strips and other floral enhancements can increase the availability of resources for beneficial arthropods and may improve biological pest-control services in adjacent crops, although effects on pollination and crop yield are more variable and depend on factors such as floral diversity, planting age, and distance from the crop [94]. Therefore, manipulating plant signals or floral resources in agricultural landscapes should be based on experimentally demonstrated receiver responses rather than on assumptions that particular colors or scents will universally attract beneficial species.

Applications of plant–animal signaling should be evaluated through measurable ecological endpoints rather than inferred directly from molecular or sensory mechanisms. In pollination studies, visitation rate and behavioral preference represent proximal responses, whereas pollen deposition, fruit set, seed set, and plant reproductive output provide stronger evidence of plant-level consequences. For seed-dispersal systems, fruit removal should ideally be linked to dispersal distance, deposition context, germination, or subsequent seedling recruitment. In herbivore-management and biological-control systems, relevant endpoints include herbivore abundance and feeding damage, predator or parasitoid recruitment, plant performance, and crop yield. At broader temporal and spatial scales, plant fitness, population persistence, and interaction-network stability provide the necessary links between individual signaling mechanisms and claims concerning conservation or ecosystem resilience. Accordingly, broad biodiversity or agricultural benefits should be inferred only when these intermediate ecological processes are demonstrated rather than extrapolated directly from molecular changes or receiver behavior.

Overall, animal perception of plant-derived information represents an interface among sensory ecology, cognition, behavior, and evolution. Plant traits can influence animal decisions only when they are detectable and biologically meaningful to the receiver, while learning and environmental context determine how that information is interpreted. Recognizing this receiver-centered perspective is essential for distinguishing true communication signals from incidental cues and for understanding how plant–animal interactions are maintained, modified, or disrupted across ecological and agricultural systems. 

### 12.5. Evolution of Signals and Receivers: Evidence for Coevolution, Exploitation, and Evolutionary Stability

Coevolution provides an important framework for interpreting plant–animal communication, but it requires stronger evidence than simple correspondence between a plant trait and an animal sensory preference. Trait matching may arise through coadaptation, sensory bias, receiver exploitation, phylogenetic constraint, or selection acting predominantly on one interacting partner. Consequently, reciprocal coevolution is most strongly supported when evolutionary change in signal production or expression can be linked to corresponding evolutionary change in receiver detection, discrimination, or response. This distinction is especially important in plant–animal systems, where close ecological association alone should not be treated as evidence of reciprocal evolutionary change.

These evolutionary processes differ in the type of evidence required for their interpretation. Coadaptation describes functional matching between interacting partners but does not necessarily imply reciprocal evolutionary change. Diffuse coevolution occurs when evolutionary responses arise from selection imposed by multiple interacting species rather than by a single pair of partners. Pollinator-mediated selection demonstrates that pollinators impose selection on floral traits, but such selection can remain unidirectional and therefore does not by itself establish coevolution. Likewise, phylogenetic correlation can reveal repeated associations or correlated trait evolution across lineages, but cannot alone demonstrate reciprocal causation. Sensory bias and receiver exploitation describe cases in which one partner evolves traits that exploit pre-existing receiver preferences. The strongest evidence for reciprocal coevolution requires direct evidence that each interacting partner imposes selection on the other and that corresponding evolutionary responses occur in both lineages.

Experimental evolution provides a useful example of the distinction between pollinator-mediated selection and reciprocal coevolution. In *Brassica rapa*, populations exposed to bumblebee versus hoverfly pollination evolved divergent floral phenotypes, including differences in floral scent emission, ultraviolet reflectance, and plant height [95]. This study provides direct experimental evidence that pollinator identity can drive adaptive evolution in plant traits. However, because corresponding evolutionary change in the pollinators was not tested, the result represents strong evidence for pollinator-mediated selection rather than demonstrated reciprocal coevolution.

Signal–receiver relationships illustrate both the potential and the limitations of coevolutionary interpretation. In insects, olfactory receptor repertoires, receptor expression, and ligand sensitivity can evolve in ways that modify the detection of ecologically important chemical information [63,65]. Such receiver diversification can facilitate increasingly specialized associations with plant-derived compounds, but receptor evolution alone does not demonstrate coevolution unless corresponding selection on plant signal production is also established. A related example occurs in plant recognition of herbivore-associated molecular patterns. Evolutionary gain and loss of functional inceptin recognition among legumes, associated with diversification of the inceptin receptor locus, demonstrates that the capacity of plants to detect herbivore-derived molecular information is itself evolutionarily labile [76,81]. However, demonstrating reciprocal coevolution additionally requires evidence that changes in plant recognition impose selection on the corresponding herbivore-derived elicitors or effectors.

Pollination systems further illustrate how reciprocal selection, reliability, and receiver behavior can shape signal evolution. Floral signals may be favored when they reliably predict rewards and are effectively learned by pollinators, potentially generating selection for signal distinctiveness and reliability [24]. In contrast, sexually deceptive orchids exploit pre-existing sensory preferences of animal receivers by mimicking components of mating signals, often without providing a nutritional reward [92]. Receiver learning and discrimination can subsequently impose selection on deceptive floral phenotypes, creating evolutionary feedback between signal production and receiver response. These systems therefore demonstrate that evolutionary change in communication may arise through mutual adaptation, conflict, or exploitation rather than through a single coevolutionary pathway.

The evolutionary stability of plant–animal communication consequently depends on the balance among signal reliability, receiver discrimination, ecological costs and benefits, and the strength and direction of selection acting on each partner. Reliable signals may be maintained when they consistently predict resources or services, whereas deceptive signals may persist when receiver discrimination is incomplete or when the fitness benefits to plants outweigh the costs imposed by receiver avoidance. Thus, coevolution in plant–animal communication is best regarded as one end of a broader evolutionary continuum that includes coadaptation, sensory exploitation, and asymmetric selection. Explicitly distinguishing among these mechanisms is necessary to avoid attributing reciprocal coevolution to interactions for which only trait association or unidirectional selection has been demonstrated.

Representative empirical studies illustrating how plant- and animal-derived information is produced, detected, and translated into behavioral or physiological responses are summarized in Table 1.

**Table 1:** Representative empirical evidence for major mechanisms of plant–animal communication.

Plant–Animal System	Signaling Mechanism	Signal or Cue Examined	Experimental/Empirical Approach	Principal Finding	Communication Level Demonstrated	Ref.
Fruit-producing plants–primate seed dispersers	Olfactory signaling	Fruit scent	Comparative chemical and ecological analyses of fruit odor in relation to dispersal strategy	Fruit scent showed patterns consistent with an evolved olfactory signal associated with primate-mediated seed dispersal	Signal production + receiver-relevant ecological association	[4]
Flowers–hawkmoths	Multimodal floral signaling	Visual and olfactory floral information	Controlled behavioral assays examining flower choice and previous experience	Hawkmoths integrated visual and olfactory information during foraging, and experience modified subsequent use of these cues	Receiver detection + multimodal integration + behavioral response	[11]
Flowers–bumblebees	Electrical signaling	Floral electric fields	Controlled discrimination and learning experiments	Bumblebees detected floral electric fields and learned to use them as additional information during flower discrimination	Receiver detection + learning + behavioral response	[16]
Plants–host-seeking insects	Volatile-mediated host location	Plant VOC blends	Atmospheric transformation experiments combined with insect behavioral assays	Chemical transformation of plant volatiles during atmospheric transmission reduced successful host-plant location	Signal transmission + receiver behavior	[22]
*Spodoptera frugiperda*–host plants	Herbivore effector signaling	Salivary effector SfPDI	Molecular, biochemical, and plant–herbivore performance assays	SfPDI modified plant defense responses in a manner that increased herbivore foraging efficiency	Animal-derived signal + altered plant physiology + ecological effect	[32]
Maize–*Spodoptera exigua*	Herbivore-associated elicitation	Volicitin in oral secretions	Chemical isolation and plant volatile-induction bioassays	Volicitin was identified as an herbivore-derived elicitor capable of inducing maize volatile emissions	Elicitor identification + plant physiological response	[34]
Bat-pollinated cactus–echolocating bats	Echo-acoustic interaction	Ultrasound-reflecting/absorbing floral architecture	Acoustic measurements of floral structures and echo contrast	A specialized ultrasound-absorbing inflorescence zone increased acoustic contrast between flowers and surrounding vegetation	Physical signal modification + enhanced receiver detectability	[55]
Tropical plants–leaf herbivores	Visual/defensive leaf coloration	Red young leaves and anthocyanins	Comparative analyses of leaf color, chemistry, and herbivore damage	Red young leaves were associated with higher anthocyanin/tannin concentrations and reduced herbivore damage, but visual signaling was not established universally	Trait association; signaling function remains conditional	[71,72]
Legumes–caterpillar herbivores	HAMP recognition	Inceptin peptides	Receptor genetics, molecular assays, and defense-response experiments	A receptor-like protein mediated recognition of caterpillar-derived inceptins and activated anti-herbivore immune responses	Molecular recognition + plant defense response	[76]
Maize–herbivore-associated airborne cues	Volatile-mediated priming	Airborne green-leaf volatiles	Volatile exposure followed by herbivore-related challenge and defense measurements	Prior exposure to airborne volatiles primed subsequent jasmonate accumulation and induced volatile responses	Signal exposure + physiological memory/priming + enhanced defense	[96]

Note: The studies shown are representative examples selected to illustrate different stages of the communication process and are not intended as an exhaustive inventory of the literature. Evidence for a communication function is strongest when signal or cue production is linked experimentally to receiver detection, a reproducible behavioral or physiological response, and an ecologically relevant consequence.

## 13. Climate Change and the Disruption of Plant–Animal Communication

Climate change can alter plant–animal communication at several interconnected stages, affecting the production of signals by plants, their transmission through the environment, and their detection and interpretation by animal receivers. Rising temperatures, altered precipitation regimes, drought, elevated atmospheric CO_2_, and increasingly frequent climatic extremes can modify plant phenology, floral metabolism, volatile emissions, visual traits, and floral rewards [97]. Because successful communication depends on both signal production and an appropriate animal response, climatic effects on either partner can modify the outcome of interactions such as pollination, seed dispersal, herbivory, and indirect plant defense [2].

For clarity, climate-related disruption can be evaluated across seven sequential but interacting stages of the communication process: (i) signal production within plant tissues; (ii) signal emission or release from the plant; (iii) transmission through the surrounding environment; (iv) detection by the animal receiver; (v) behavioral interpretation and decision-making; (vi) temporal synchrony between plant and animal activity; and (vii) ecological consequences for plant reproduction, herbivore resistance, natural-enemy recruitment, or population-level dynamics. Environmental change may affect one or several of these stages simultaneously, and evidence at one stage should not be assumed to demonstrate disruption across the entire communication pathway.

One of the most consistently documented consequences of contemporary climate change is altered phenology. Plants and animals do not necessarily adjust the timing of their seasonal activities at the same rate, creating the potential for temporal asynchrony between flowering or fruiting and the activity of pollinators, dispersers, or herbivores [93]. A recent global analysis of nearly half a million terrestrial phenological time series found increasing phenological divergence between plants and animals. Covering the period 1981–2020, the analysis further showed that almost 30% of temporal variation in plant phenophases could be explained by the timing of preceding phenophases, suggesting that shifts in one phenological event can propagate to subsequent seasonal events and that continued warming may further alter trophic synchrony [93]. Earlier work on plant–pollinator systems similarly demonstrated that warming can alter both phenology and geographic distributions, although the magnitude and direction of responses vary among species [98]. Thus, climate-driven disruption should not be interpreted simply as an earlier flowering date, but as a potential change in the temporal overlap between interacting organisms.

The effects of climate change also extend directly to signal production and emission. Heat and water stress can modify flower development, floral rewards, pigmentation, VOC biosynthesis, and the quantity or composition of volatiles released from floral tissues [97,99]. Environmental stress can also alter the physiological state of plants through changes in ionic balance and oxidative status. For example, zinc nutrition has been reported to enhance plant tolerance to salinity by improving ionic homeostasis and antioxidant defenses [100]. Such physiological responses provide an important background for understanding how environmental stress may subsequently influence plant traits involved in ecological interactions.

Quantitative experiments illustrate the magnitude of these effects. In *Borago officinalis*, comparing well-watered plants at 21 °C with water-stressed plants at 27 °C, flower number decreased from 15 ± 2 to 8 ± 1 flowers per plant, corolla area declined from 3.8 ± 0.5 to 2.2 ± 0.1 cm^2^, nectar sugar content fell from 3.9 ± 0.3 to 1.3 ± 0.4 mg per flower, and pollen viability decreased from 79 ± 4% to 25 ± 9%; flowers under the unstressed treatment also received at least twice as many bumblebee visits [99]. Importantly, responses are not uniformly negative. In a four-species forb experiment, drought reduced petal area by 18–32% in some species while increasing total floral VOC emissions by 98–129%, and pollinator visitation to drought-treated *Potentilla recta* was 52% lower than to controls [101]. These results show that climate stress can simultaneously weaken visual or reward-related traits while increasing some olfactory emissions, making the net communication outcome strongly context dependent, for example, altering floral scent profiles and pollinator visitation, while effects on nectar production differed among plant species [9]. Elevated CO_2_ can likewise modify flowering phenology and floral traits, but available evidence does not support a universal reduction in pollinator visitation or plant reproductive success [102]. These context-dependent responses are important because changes in signal composition may affect communication even when total signal production does not decline.

Changes in the physical environment can additionally affect the transmission of signals between plants and animals. These transmission and degradation processes are conceptually distinct from changes in VOC biosynthesis or emission occurring at the plant source. Floral VOCs are particularly vulnerable because they must travel through the atmosphere before reaching an animal receiver. Atmospheric oxidants such as ozone can react with floral volatiles, alter the relative proportions of compounds within scent blends, and reduce the distance over which flowers remain olfactorily detectable. In *Brassica nigra*, floral VOC degradation became detectable at 80 ppb O_3_ over 1.5 m, while exposure to 120 ppb O_3_ over 4.5 m reduced the concentrations of several floral volatiles by approximately 25–30% and significantly reduced attractiveness to *Bombus terrestris* [103]. Field-scale experiments further showed that diesel exhaust and ozone reduced pollinator abundance by up to 48% and parasitoid abundance by up to 32% [104]. Although air pollution is not itself synonymous with climate change, these findings demonstrate how interacting anthropogenic pressures can alter the transmission and biological effectiveness of chemical signals. Viewed through the multimodal framework developed in this review, different environmental drivers affect distinct but interacting communication channels. Phenological shifts primarily alter the temporal availability of multiple modalities by changing whether plant signals and animal receivers overlap in time [93,98]. Warming and drought can modify visual traits, floral rewards relevant to gustatory evaluation, and chemical signals such as VOCs [97,99,101], whereas elevated CO_2_ can alter both visual and olfactory floral traits and their relationship with pollinator visitation [101,102]. Ozone and diesel exhaust act particularly strongly on chemical communication by degrading or modifying airborne scent information and reducing receiver activity [103,104]. Acoustic and vibrational channels may also be vulnerable to environmental change through altered background noise and changes in physical transmission conditions; however, current evidence for climate-specific disruption of these modalities remains less developed than for chemical and phenological pathways [48,54,55]. Thus, environmental change should be understood as a multimodal disruption process rather than as an effect on any single signaling channel. Figure 4 summarizes these mechanisms by distinguishing effects on the plant signal source, the transmission environment, and the animal receiver.

Animal receivers are also directly affected by changing environmental conditions. Temperature influences insect development, seasonal activity, flight, and foraging behavior and can therefore alter when and how animals encounter plant-derived signals [98,99]. Environmental stress can consequently disrupt communication even when a plant continues to produce a recognizable signal. At the same time, altered floral rewards can change the behavioral value associated with plant signals. For example, experimental increases in temperature and water stress in *Borago officinalis* reduced several floral traits and nectar sugar production and were accompanied by substantial reductions in bumblebee visitation [99]. Such results illustrate that climate effects on plant–animal communication often arise from simultaneous changes in signal phenotype, reward availability, and receiver behavior.

Spatial changes provide another potential source of disruption. As climatic conditions shift, plant and animal distributions may change at different rates, altering the probability that historically interacting species continue to encounter one another [98]. Range shifts can also create interactions among species without a long history of association. Such novel encounters should not automatically be assumed to be dysfunctional, because generalist animals may readily exploit unfamiliar resources. Nevertheless, changes in community composition can alter the sensory and ecological context in which signals are produced and interpreted, potentially reorganizing interaction networks.

The combined consequences of temporal, spatial, and sensory changes can therefore extend beyond individual plant–animal pairs. Reduced overlap between plants and pollinators may modify pollination opportunities, while changes in animal distributions may alter seed-dispersal pathways and herbivore communities. Abiotic stress can also influence herbivore-induced plant volatiles and the recruitment of predators and parasitoids, although the direction and magnitude of these effects vary among systems [105]. This variability is particularly important for biological control because plant–natural enemy communication can be maintained under some environmental stresses but disrupted under others [105]. Field evidence that atmospheric pollution disproportionately reduces pollinator and parasitoid activity further demonstrates that anthropogenic environmental change can modify different functional groups within the same ecological community [104].

These ecological pathways are summarized in Figure 5, which extends the mechanistic framework of Figure 4 to the broader consequences of communication disruption.

The strength of evidence nevertheless differs among stages and mechanisms. Evidence is strongest for climate-related shifts in phenology and for experimentally demonstrated changes in floral traits, rewards, VOC emissions, atmospheric scent degradation, and pollinator or parasitoid behavior [93,99,101,103,104]. Evidence for the direction of drought- or CO_2_-mediated changes in communication is more variable, because responses differ among plant species, animal receivers, and environmental conditions [9,101,102]. In contrast, broader consequences for long-term population persistence, community reorganization, and climate-specific disruption of acoustic or vibrational communication remain less directly demonstrated and should presently be regarded as emerging or partly hypothetical rather than established general effects [48,54,55].

The agricultural consequences of these processes deserve particular attention. Many crops depend on animal pollination, while natural enemies recruited directly or indirectly through plant-associated cues contribute to biological pest control. Climate-induced changes in floral traits, reward availability, signal transmission, and animal activity could therefore alter ecosystem services even before complete species loss occurs [97,99]. However, responses are unlikely to be uniform across crop species, pollinator communities, or climatic regions. Elevated CO_2_, for example, can shift flowering phenology without necessarily reducing total visitation or seed production [102], while drought effects on scent and visitation may differ among co-occurring plant and pollinator species [9]. Predictions should therefore be based on specific mechanisms and ecological contexts rather than on the assumption that all components of climate change uniformly weaken communication.

Overall, climate change can reshape plant–animal communication by altering when signals are produced, what information they contain, how effectively they are transmitted, and whether animal receivers are present and capable of responding to them. The resulting effects range from subtle changes in signal reliability to temporal and spatial mismatches and, ultimately, reorganization of ecological networks. Understanding these processes will require integration of plant physiology, chemical and sensory ecology, animal behavior, phenology, and community ecology. Such an approach is essential for predicting which plant–animal interactions are most vulnerable and for developing conservation and agricultural strategies capable of maintaining pollination, seed dispersal, and biological control under ongoing environmental change.

## 14. Genetic Manipulation of Plant Signaling: Opportunities and Ecological Consequences

Agricultural manipulation of plant signaling can be achieved through approaches that differ substantially in mechanism, precision, persistence, and ecological implications. Conventional breeding acts through selection and recombination of naturally occurring genetic variation and generally modifies multiple linked traits over successive generations. Transgenic approaches introduce or ectopically express genetic material and can generate novel or substantially altered metabolic phenotypes. Genome editing enables more targeted modification of endogenous genes or regulatory regions, although precise molecular editing does not necessarily imply ecological specificity because edited pathways may have pleiotropic effects. Exogenous manipulation, including application of jasmonates, elicitors, or other signaling compounds, does not necessarily alter the plant genome and is typically more transient and dependent on dose, timing, environmental exposure, and compound persistence. These approaches should therefore not be treated as ecologically equivalent, even when they target the same hormonal, volatile, or specialized-metabolite pathway [106,107]. These intervention strategies are also not equivalent from a regulatory perspective, because conventional breeding, transgenic modification, genome editing, and exogenous chemical treatments may be subject to different biosafety, environmental-risk-assessment, and regulatory requirements depending on the jurisdiction and the nature of the resulting phenotype. Exogenous induction also provides an experimentally tractable alternative to permanent genetic modification. In field-grown tomato, application of jasmonic acid increased parasitism of herbivorous caterpillars approximately twofold, demonstrating that externally induced signaling can alter multitrophic interactions under agricultural field conditions [108]. However, such responses depend on treatment concentration, timing, plant developmental stage, and environmental conditions, and therefore cannot be assumed to reproduce the consequences of constitutive genetic modification.

Phytohormone and epitranscriptomic pathways are best considered here as upstream regulatory contexts rather than as direct evidence of plant–animal communication. Jasmonate signaling is directly relevant because it regulates a wide range of chemical and structural defenses against herbivores and pathogens [109], thereby providing a mechanistic route through which regulatory changes can alter traits encountered by animal receivers. By contrast, manipulation of brassinosteroid signaling has primarily been examined for effects on plant architecture, reproductive development, and stress adaptation [110]. Such changes become relevant to plant–animal communication only if they subsequently modify animal-facing phenotypes, such as floral display, pigmentation, volatile production, or other traits that influence receiver detection and behavior [2,3,107]. A similar distinction applies to N^6^-methyladenosine (m^6^A) regulation. m^6^A interacts extensively with plant hormone pathways and contributes to the regulation of development and environmental responses [111], while disruption of the m^6^A regulatory machinery can alter photosynthetic performance under high light stress [112]. These examples therefore illustrate upstream regulatory leverage and potential pleiotropic consequences, rather than direct evidence that brassinosteroid or m^6^A manipulation improves plant–animal communication. Demonstrating such a connection requires showing that the regulatory intervention changes an animal-relevant signal phenotype and that the altered phenotype is detected and used by an animal receiver. In contrast to these upstream regulatory examples, volatile organic compounds provide a more direct connection between genetic manipulation and plant–animal communication. Plant VOCs contribute to pollinator attraction, host recognition, herbivore deterrence, and the recruitment of predators and parasitoids, making volatile biosynthetic pathways attractive targets for metabolic engineering [107]. Experimental genetic manipulation of terpenoid metabolism in *Arabidopsis thaliana*, for example, generated plants that emitted novel terpenoid volatiles and attracted the predatory mite *Phytoseiulus persimilis*, demonstrating that modification of a plant metabolic pathway can alter the behavior of an animal receiver [113]. Such results provide direct evidence that engineered changes in plant chemistry can modify multitrophic interactions rather than merely altering internal plant physiology.

Regulatory manipulation can also change plant attractiveness to herbivores. In rice, overexpression of the ethylene-signaling transcription factor *OsEIL1* altered the expression of several terpene synthase genes, including down-regulation of the limonene synthase gene *OsTPS19*. The resulting reduction in limonene production and emission contributed to increased attractiveness of the plants to female brown planthoppers for feeding and oviposition [114]. This example is particularly relevant because it demonstrates that genetic modification of a hormonal signaling pathway can indirectly alter volatile production and subsequently change insect behavior. Thus, signaling pathways that appear distinct at the molecular level can become ecologically interconnected through their effects on plant chemical phenotypes.

Specialized metabolites may introduce additional complexity because individual compounds can participate simultaneously in defense, stress responses, and interactions with other organisms. Phenolics, flavonoids, terpenoids, and related metabolites are therefore potential targets for metabolic engineering, but their ecological functions cannot be predicted solely from their concentrations. For example, β-caryophyllene and eugenol can alter the expression of thousands of genes in *Arabidopsis*, including genes associated with hormone signaling and cell-wall metabolism [115]. Although that study involved exposure to these compounds rather than genetic modification, it illustrates the broad regulatory consequences that changes in specialized-metabolite pathways could potentially generate. Accordingly, engineered increases or decreases in a particular metabolite may affect several physiological processes simultaneously rather than producing a single isolated signaling effect.

The principal pathways through which genetic manipulation may influence plant signaling and plant–animal interactions are summarized in Figure 6.

The ecological effects of engineered signaling traits are not necessarily uniform across animal species. Modification of plant volatile emissions can produce different responses in herbivores, predators, and parasitoids. In genetically engineered *Arabidopsis* lines with altered volatile terpenoid production, for example, behavioral responses differed among an aphid herbivore, a parasitoid, and a predator [116]. These receiver-specific responses demonstrate why changes that enhance one ecological service, such as recruitment of a parasitoid, cannot automatically be assumed to improve the entire interaction network. Similarly, metabolic engineering of VOC pathways may generate unexpected changes in volatile composition or other metabolic traits because individual compounds and biosynthetic pathways often perform multiple physiological and ecological functions [107].

Ecological risk assessment of manipulated signaling traits must extend beyond the intended target organism. Altered volatile or specialized-metabolite profiles may affect non-target herbivores, pollinators, predators, parasitoids, and other arthropods that share the same chemical environment. Experimental work with engineered terpenoid-emitting plants already demonstrates that different herbivores, predators, and parasitoids can respond differently to the same modified volatile phenotype [116]. At the community level, changing the identity, concentration, or ratio of emitted compounds may also alter plant–plant chemical interactions and the wider semiochemical network through which neighboring plants and multiple animal receivers obtain information [87,107]. Consequently, an intervention that improves attraction of one beneficial organism may simultaneously alter pollinator behavior, herbivore attraction, or natural-enemy recruitment in unintended ways.

The temporal and evolutionary dimensions of risk also differ among intervention types. Heritable modifications introduced through breeding, transgenesis, or genome editing can persist across generations and may impose sustained selection on associated insect populations, whereas exogenous treatments are generally more transient but may still have exposure- and persistence-dependent ecological effects. Prolonged deployment of strongly selective traits can favor evolutionary responses in herbivore populations, including resistance or behavioral avoidance, as demonstrated broadly in insect adaptation to transgenic insecticidal crops [117]. Laboratory assays are therefore insufficient for predicting long-term ecological performance: effects observed under controlled conditions may change under field variation in plant genotype, climate, background odors, community composition, natural-enemy abundance, and insect evolutionary responses. Long-term field validation should consequently include both efficacy and unintended ecological effects.

From an agricultural perspective, breeding, transgenic modification, genome editing, and exogenous manipulation of plant signaling all offer opportunities to strengthen herbivore resistance, improve stress resilience, and potentially enhance recruitment of beneficial organisms. Nevertheless, successful application requires more than demonstrating altered gene expression, metabolite accumulation, or short-term behavioral responses. Modified or treated plants should be evaluated for changes in signal composition, animal detection and behavior, effects on target and non-target organisms, persistence of the induced phenotype or treatment, and consequences at multitrophic and ecosystem levels [107,116]. This is particularly important for volatile-mediated communication, where small changes in compound identity, concentration, or blend ratios may produce different behavioral outcomes among animal receivers.

Overall, manipulation of plant signaling through breeding, transgenic approaches, genome editing, or exogenous treatments can provide powerful tools for investigating and modifying plant–animal interactions, but ecological outcomes are inherently context dependent. The most informative approaches will therefore combine molecular genetics and metabolic engineering with behavioral assays, chemical ecology, and ecological validation. Such integration can help distinguish genetic modifications that genuinely enhance desirable plant–animal interactions from those that generate unintended changes in receiver behavior or ecological network structure. The strength of evidence is not uniform across the different components of plant–animal communication. 

Table 2 applies this common evidence chain across the principal mechanisms considered in the review by distinguishing signal or cue production, receiver detection, behavioral or physiological response, plant-level benefits or costs, evolutionary interpretation, and validation under natural or ecologically realistic conditions. The table also identifies where individual links in this chain remain incomplete, thereby distinguishing well-supported communication functions from candidate cues or traits for which evidence remains partial.

**Table 2:** Evidence-chain assessment of major mechanisms and themes in plant–animal communication.

Theme or Mechanism	Signal/Cue Production or Modification	Receiver Detection	Behavioral/Physiological Response	Plant benefit, Cost, or Ecological Consequence	Evolutionary Interpretation	Evidence Under Natural/Ecologically Realistic Conditions	Overall Synthesis/Representative Refs.
Plant VOCs in host location and foraging	Dynamic VOC production and variation in blend composition are well established.	Strong olfactory and behavioral evidence shows that insects detect plant VOCs and blend composition.	VOCs alter host location, foraging, pollinator attraction, and natural-enemy responses.	Effects may benefit plants through pollination or natural-enemy recruitment, but may be costly when herbivores use the same information to locate hosts.	Signal adaptation and receiver exploitation are context dependent; reciprocal selection should not be assumed universally.	Supported by laboratory, semi-field, and field studies, although atmospheric and community context can modify responses.	Strong [18,20,22,23,59,64]
Multimodal floral signaling	Visual, olfactory, morphological, and reward-associated floral traits are well established.	Pollinators can detect and integrate several sensory components.	Integrated cues influence flower choice, learning, and foraging decisions.	Increased detectability or efficient resource localization may enhance visitation and pollination, although fitness consequences are not always measured.	Pollinator-mediated selection and receiver learning can shape floral traits, but reciprocal coevolution is not demonstrated in every system.	Controlled behavioral evidence is strong; ecological and fitness validation varies among systems.	Strong [11,24,88,89,91]
Floral electric fields	Electric fields associated with flowers are experimentally measurable.	Bumblebees can detect differences in floral electric fields.	Bees can learn associations between electric fields and floral information and use them in discrimination.	Potential effects on flower choice are demonstrated, but plant reproductive consequences remain insufficiently quantified.	Evidence for adaptive or reciprocal evolutionary change remains limited.	Evidence is derived mainly from controlled experimental systems; population- and community-level validation is limited.	Moderate–Strong [16]
Herbivore-derived HAMPs and effectors	Herbivore-derived elicitors and effectors occur in saliva, oral secretions, regurgitant, and feeding-associated materials and modify plant signaling.	Direct plant recognition has been demonstrated for selected HAMPs.	Recognition activates or redirects plant defense signaling and physiology.	Outcomes include altered resistance, herbivore performance, systemic defense, and recruitment of natural enemies.	Antagonistic coadaptation is plausible, and evolutionary gain/loss of recognition is documented in selected systems; reciprocal selection requires case-specific evidence.	Strong mechanistic evidence exists, with ecological validation available for selected plant–herbivore systems.	Strong for characterized systems [20,31,32,33,34,35,75,76,77,81,82,96]
Semiochemicals and olfactory reception	Plant VOCs, pheromones, and other semiochemicals are chemically characterized in many systems.	Extensive receptor and sensory-neuron evidence supports discrimination.	Chemical information alters host choice, mate location, foraging, and ecological eavesdropping.	Consequences can include pollination, herbivory, natural-enemy recruitment, or exploitation of the emitter.	Receiver exploitation and eavesdropping are common; adaptive and coevolutionary interpretations depend on the specific interaction.	Behavioral and field evidence is available in multiple systems, but ecological function remains context dependent.	Strong [5,6,57,58,59,60,61,62,63,64,65,66]
Leaf coloration as a visual signal	Pigment production and developmental or seasonal color changes are well documented.	Animal ability to discriminate leaf colors varies among taxa.	Behavioral responses to coloration are demonstrated in some systems but remain weak or absent in others.	Associations with herbivory and defense exist, but physiological functions may occur independently of signaling.	Adaptive signaling and coevolutionary explanations remain debated because photoprotection and other physiological alternatives exist.	Comparative field evidence exists, but direct demonstrations linking perception to plant fitness remain limited.	Context dependent/Moderate [67,68,69,70,71,72,73,74]
Plant-generated sounds as putative animal-directed signals	Physiological and mechanical sound production by plants has been demonstrated.	Consistent detection and use by animal receivers remain insufficiently demonstrated.	Reproducible animal responses to plant-generated sounds are not established broadly.	Direct fitness or community-level benefits to plants remain unresolved.	Evidence for evolved animal-directed acoustic signaling or reciprocal selection is currently lacking.	Ecologically realistic demonstrations remain very limited.	Emerging/Limited [8,50,51,52,53]
Echo-acoustic plant traits in bat mutualisms	Floral and fruit morphology can modify returning ultrasonic echoes.	Echolocating bats detect and use acoustic contrast.	Echo-acoustic properties can improve localization of flowers or fruits.	Improved localization may facilitate pollination or seed dispersal in specialized systems.	Specialized morphology is consistent with adaptation to bat sensory systems, although reciprocal evolutionary evidence remains system-specific.	Supported by experiments conducted in biologically relevant bat–plant systems, but taxonomic generality is limited.	Moderate–Strong [17,54,55,56]
Climate-driven disruption of communication	Climate drivers can modify phenology, VOCs, floral traits, rewards, and signal transmission.	Receiver activity and signal detectability can change under altered environmental conditions.	Changes in visitation, attraction, foraging, and temporal overlap have been demonstrated.	Consequences include altered pollination, herbivory, natural-enemy interactions, and reproductive outcomes.	Climate change can alter selective environments, but observed responses should not themselves be interpreted as coevolution.	Field observations, long-term datasets, and experimental studies provide direct evidence, but effects are highly species and context dependent.	Moderate–Strong, context dependent [93,97,98,99,101,102,103,104,105]
Genetic manipulation of plant signaling	Gene editing, transgenic manipulation, gene silencing, and metabolic engineering can alter signaling and specialized metabolism.	Animal receivers respond to some experimentally altered volatile phenotypes.	Modified plants can change herbivore, predator, parasitoid, or pollinator behavior.	Benefits to target interactions may be accompanied by costs to non-target organisms or multitrophic networks.	These are experimentally imposed changes and should not be interpreted as evidence of natural adaptation or coevolution.	Most evidence remains experimental; long-term field and community-level validation is comparatively limited.	Moderate [106,107,109,110,111,112,113,114,115,116]

Evidence-assessment framework: Strong indicates convergent direct evidence linking the biological source of information with receiver detection or response and an ecologically relevant consequence across multiple studies or systems. Moderate–Strong indicates direct mechanistic and/or behavioral evidence with more limited taxonomic or ecological replication. Moderate indicates credible experimental evidence but incomplete linkage across the full communication chain or limited ecological generality. Emerging/Limited indicates that the underlying phenomenon has been demonstrated, but evidence for receiver-mediated communication and ecological function remains insufficient. Context dependent indicates that alternative physiological functions or substantial variation among species prevents a general signaling interpretation. These categories represent a qualitative critical synthesis of the literature reviewed here and are not a formal systematic-review or meta-analytic grading system. Importantly, the overall synthesis category summarizes but does not replace the component-level evidence assessment; laboratory detection or behavioral discrimination alone is not considered sufficient evidence of ecological function, adaptive signaling, or reciprocal evolutionary change.

## 15. Conclusions

Plant–animal communication encompasses diverse forms of information transfer that may operate through chemical, visual, gustatory, electrical, and acoustic or vibrational channels, either independently or, where receiver integration is demonstrated, multimodally. The effectiveness of these interactions depends not only on signal production but also on transmission, receiver perception, learning, and ecological context. Importantly, detectable plant traits should not automatically be interpreted as evolved signals; demonstrating communication requires evidence that the information is perceived by an appropriate receiver and produces a biologically meaningful response. Environmental change can disrupt these relationships by altering phenology, signal composition, transmission, and animal sensory responses, potentially reorganizing pollination, seed dispersal, herbivory, and biological control networks. Genetic manipulation of plant signaling may provide opportunities to modify defense, volatile production, and stress responses, but its ecological consequences should be evaluated across animal receivers and multitrophic interactions. Future research should therefore integrate plant molecular biology, sensory ecology, animal behavior, and environmental science to determine how signaling mechanisms operate under realistic ecological conditions. Such integration will be essential for understanding the resilience of plant–animal interactions and for supporting biodiversity conservation, sustainable pollination, and ecological pest management. Across the evidence base synthesized here, chemical and visual interactions are supported by the broadest combination of mechanistic, behavioral, and ecological evidence, whereas electrical and plant-generated acoustic or vibrational phenomena remain more system-specific and are often incomplete at later stages of the evidence chain. Reciprocal coevolution should likewise be inferred only where reciprocal selection or corresponding evolutionary change is demonstrated in both interacting lineages. Major unresolved questions therefore concern the ecological generality of emerging sensory channels, the extent of true multimodal integration under natural conditions, and the long-term fitness and community consequences of environmentally altered or experimentally manipulated signaling traits.

Accordingly, five research priorities emerge:

(1) Field validation of cue-versus-signal claims. Putative communication traits should be tested under realistic ecological conditions to determine whether they are consistently detected by intended receivers, produce reproducible behavioral or physiological responses, and generate measurable ecological or fitness consequences.
(2) Receiver-centered mapping of plant traits. Future studies should explicitly link plant signal properties to the sensory capacities, detection thresholds, learning, and decision-making processes of relevant animal receivers rather than inferring communication from trait conspicuousness or chemical presence alone.
(3) Experimental dissection of multimodal interactions. Factorial experiments manipulating visual, olfactory, gustatory, electrical, and acoustic or vibrational components independently and in combination are needed to distinguish additive, synergistic or configural, redundant or complementary, antagonistic, sequential, and context-dependent effects on receiver behavior.
(4) Long-term monitoring of environmental disruption. Coordinated phenological, sensory, and ecological-network monitoring should be used to determine how climate change, atmospheric pollution, habitat alteration, and other environmental pressures modify signal production, transmission, receiver activity, and interaction stability over time.
(5) Ecological risk assessment of engineered signaling traits. Genetic or metabolic manipulation of volatile and other signaling pathways should be evaluated not only for intended effects on target organisms but also for consequences for non-target pollinators, herbivores, predators, and parasitoids, multitrophic interactions, and the potential for evolutionary responses in receiver populations.

## Figures and Tables

**Figure 1:**
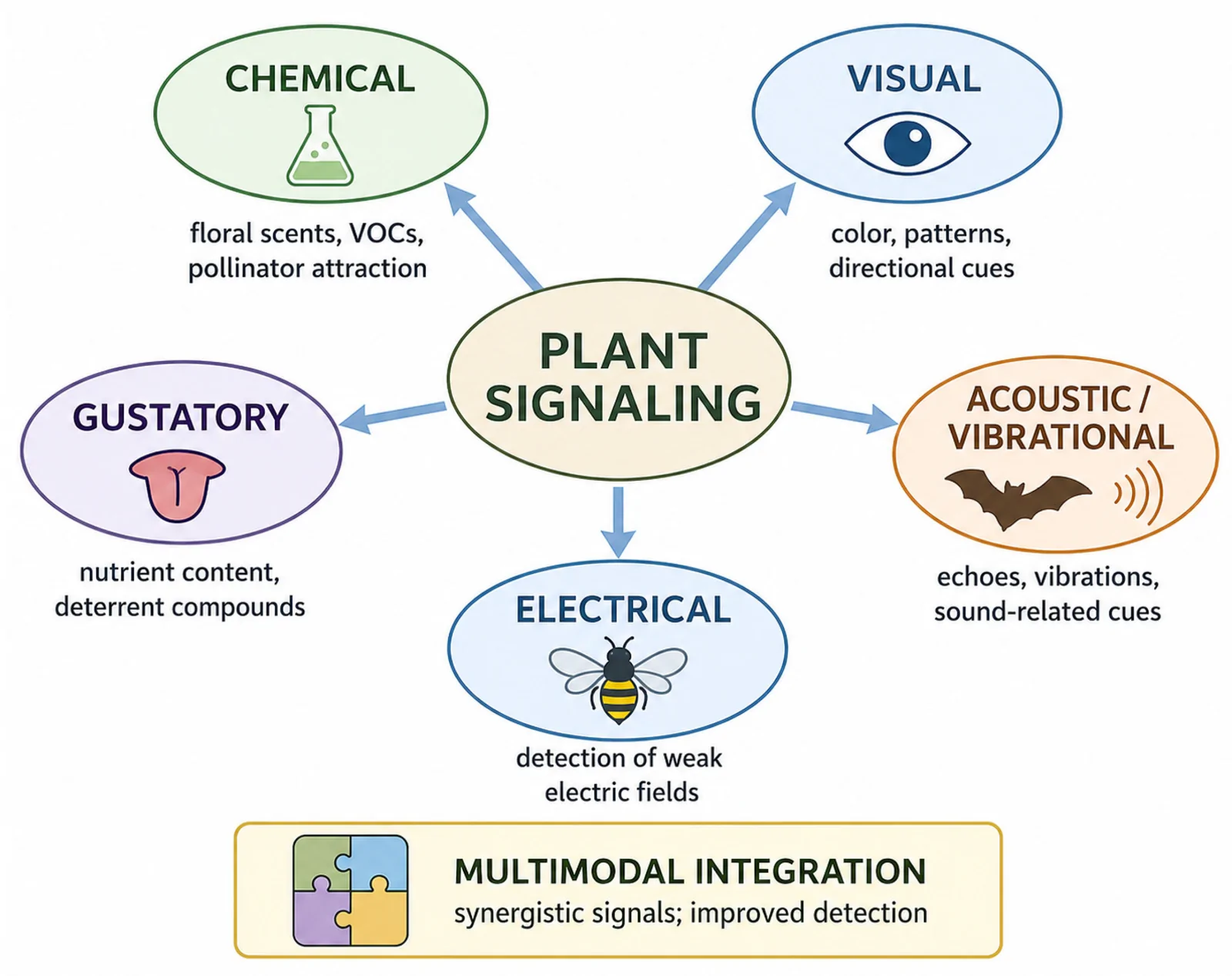
Major signaling modalities involved in plant–animal communication. Plants can provide chemical, visual, gustatory, and electrical information detectable by animal receivers, whereas acoustic and vibrational interactions range from well-supported passive or substrate-mediated processes to emerging plant-generated phenomena for which receiver-mediated communication remains incompletely demonstrated. These sensory channels may act independently or be integrated as multimodal signals, thereby influencing ecological processes such as pollination, seed dispersal, host location, and herbivore deterrence. Electrical and acoustic/vibrational modalities are included as emerging or system-specific channels for which the evidence base is less extensive than for chemical and visual communication.

**Figure 2:**
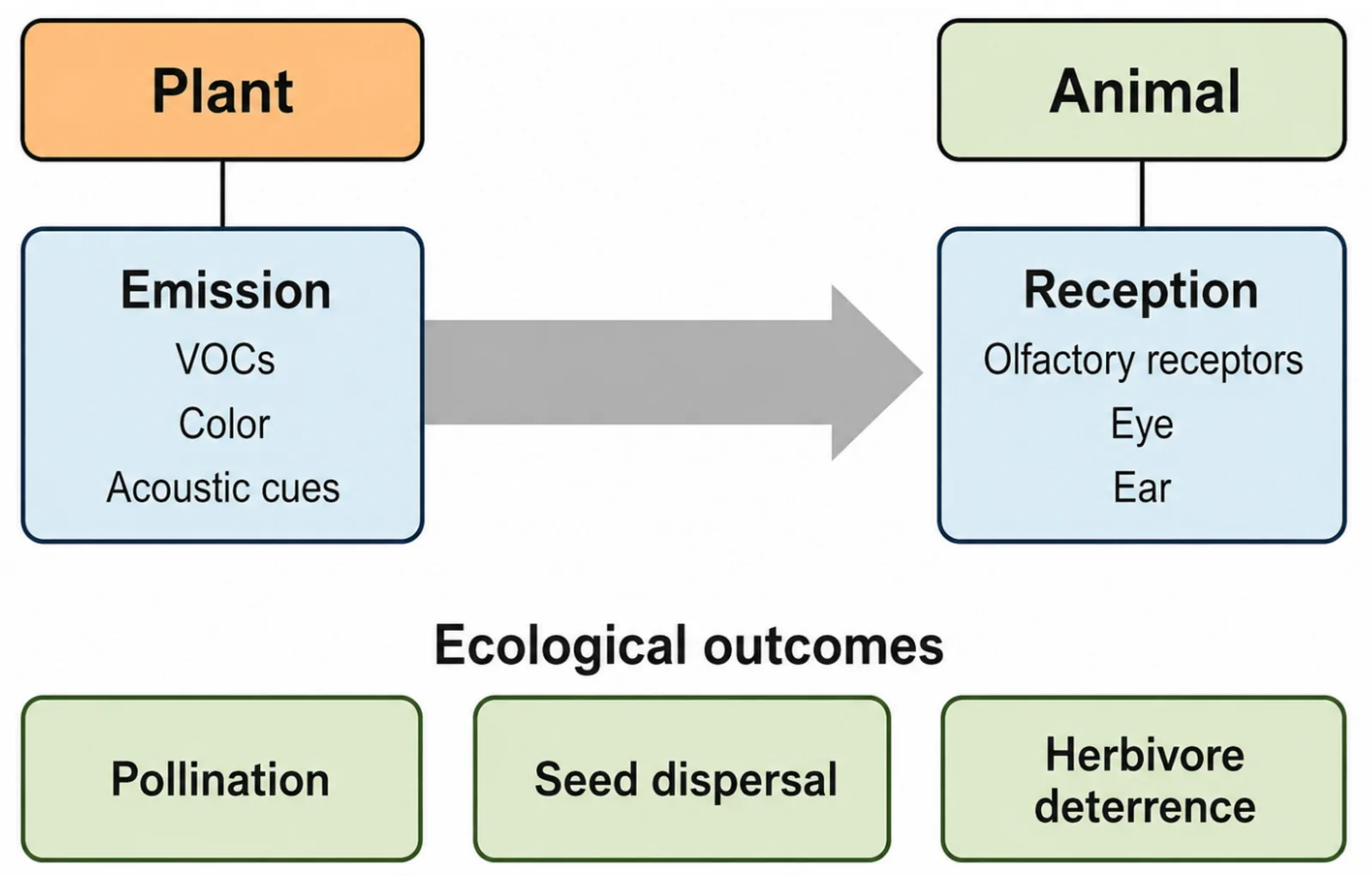
Conceptual representation of signal emission, reception, and ecological outcomes in plant–animal communication. Plants generate or express diverse sources of information, including volatile organic compounds (VOCs), visual traits, and acoustic information, which may function as cues or signals depending on receiver use and the available evidence for ecological and evolutionary function. These sources of information can be detected by animals through corresponding sensory systems such as olfactory receptors, vision, and hearing. The perception and interpretation of this information can influence animal behavior and contribute to major ecological outcomes, including pollination, seed dispersal, and herbivore deterrence.

**Figure 3:**
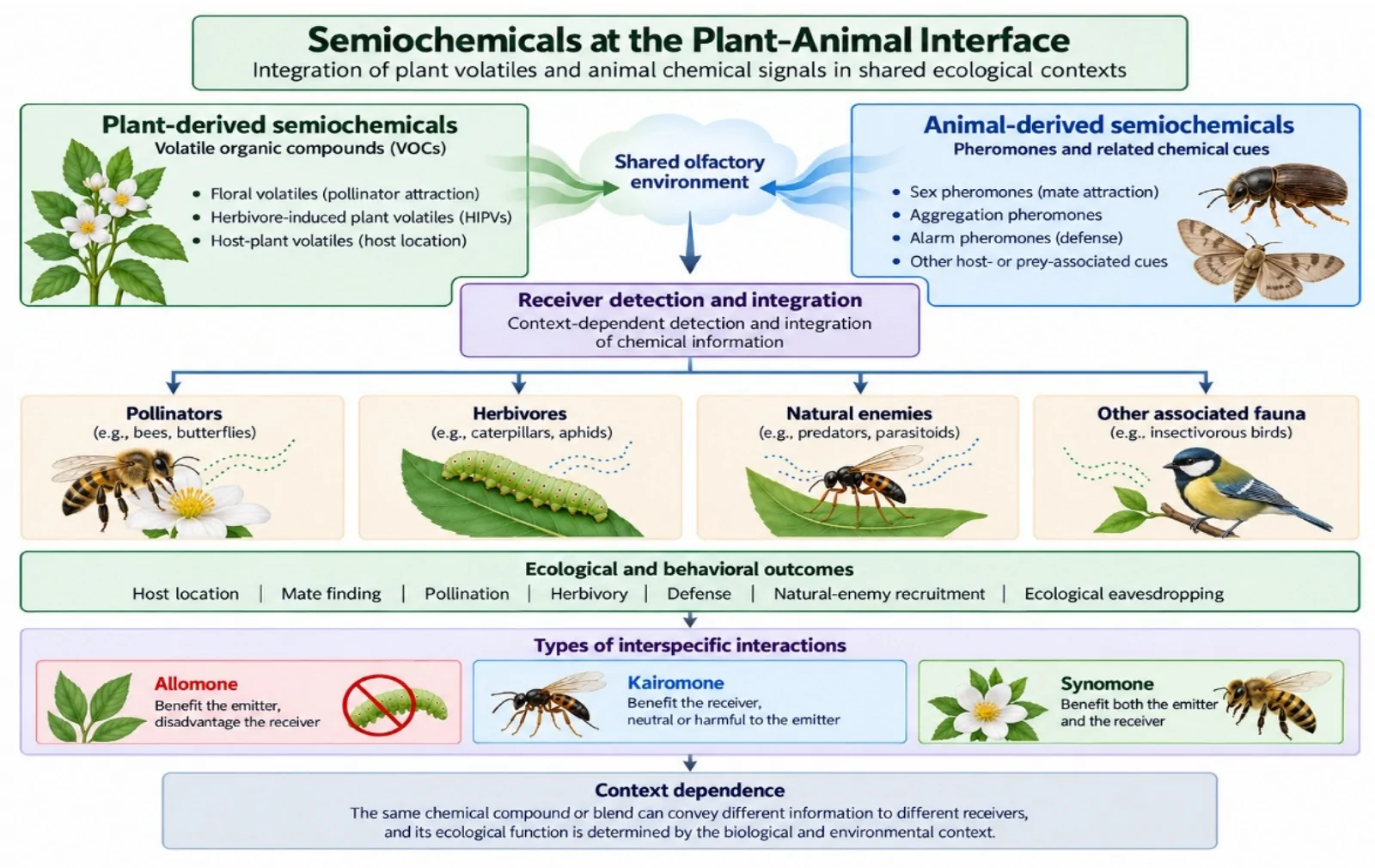
Semiochemicals at the plant–animal interface. Plant-derived volatile organic compounds (VOCs) and animal-derived chemical signals occur within a shared olfactory environment and may be detected by multiple receivers. Interactions among these chemical sources can influence pollinators, herbivores, natural enemies, and other trophic levels. Depending on the ecological consequences for emitter and receiver, interspecific semiochemicals may function as allomones, kairomones, or synomones. Ecological eavesdropping further allows unintended receivers, including predators and parasitoids, to exploit chemical information produced in other interactions.

**Figure 4:**
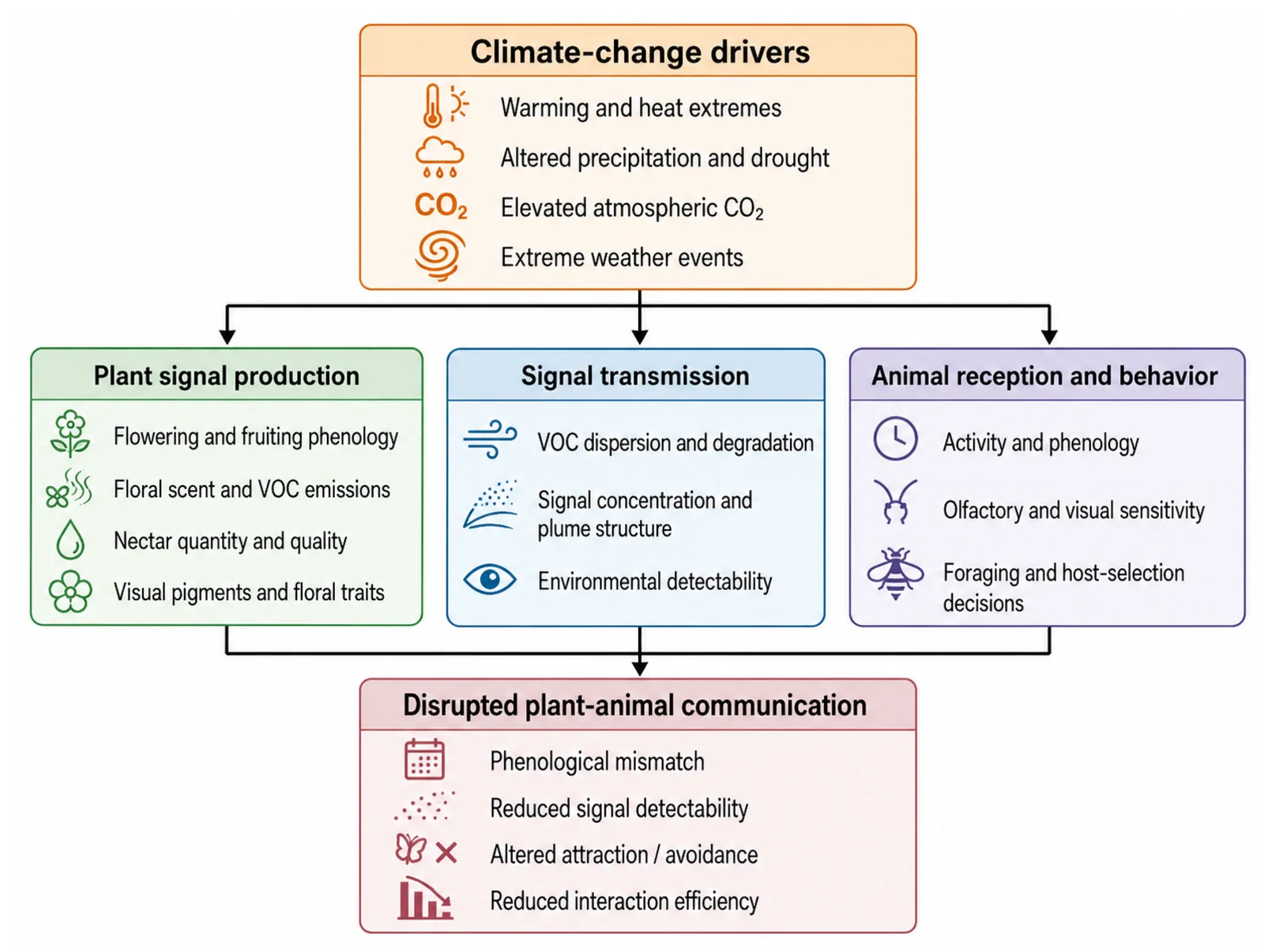
Conceptual framework illustrating the effects of climate change on plant–animal communication. Climate-change drivers, including warming and heat extremes, altered precipitation and drought, elevated atmospheric CO_2_, and extreme weather events, can influence signal production and emission, environmental transmission, animal sensory detection and behavioral interpretation, and plant–animal phenological synchrony. These disruptions may reduce signal detectability, alter attraction or avoidance responses, generate temporal mismatches between interacting species, and ultimately affect ecological outcomes such as pollination, seed dispersal, herbivore resistance, natural-enemy recruitment, and the efficiency of plant–animal interactions.

**Figure 5:**
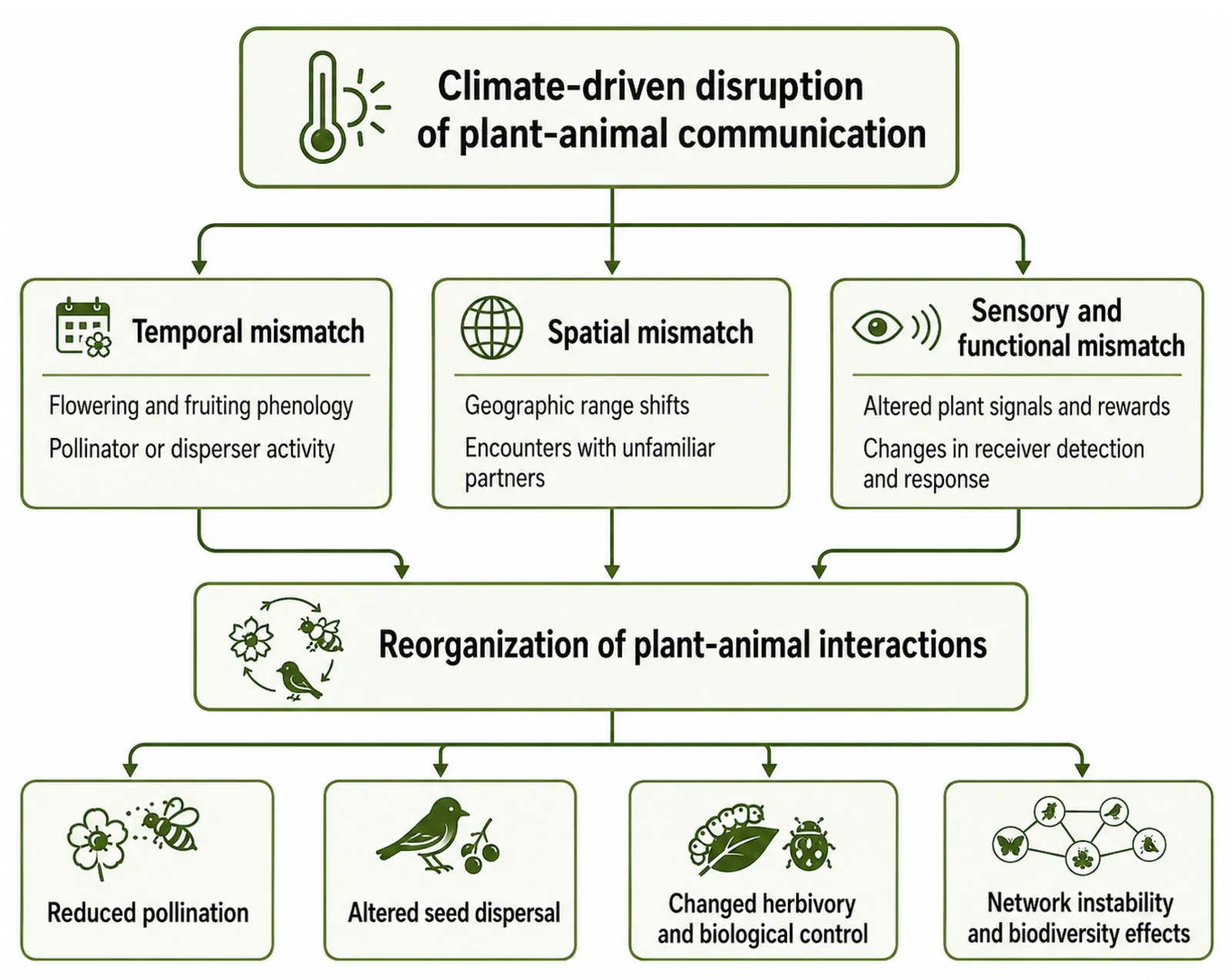
Ecological consequences of climate-driven disruption of plant–animal communication. Climate-driven disruption of plant–animal communication can generate temporal, spatial, and sensory or functional mismatches between plants and their animal partners. These mismatches may reorganize plant–animal interactions and contribute to reduced pollination, altered seed dispersal, changed herbivory and biological control, and broader effects on ecological network stability and biodiversity.

**Figure 6:**
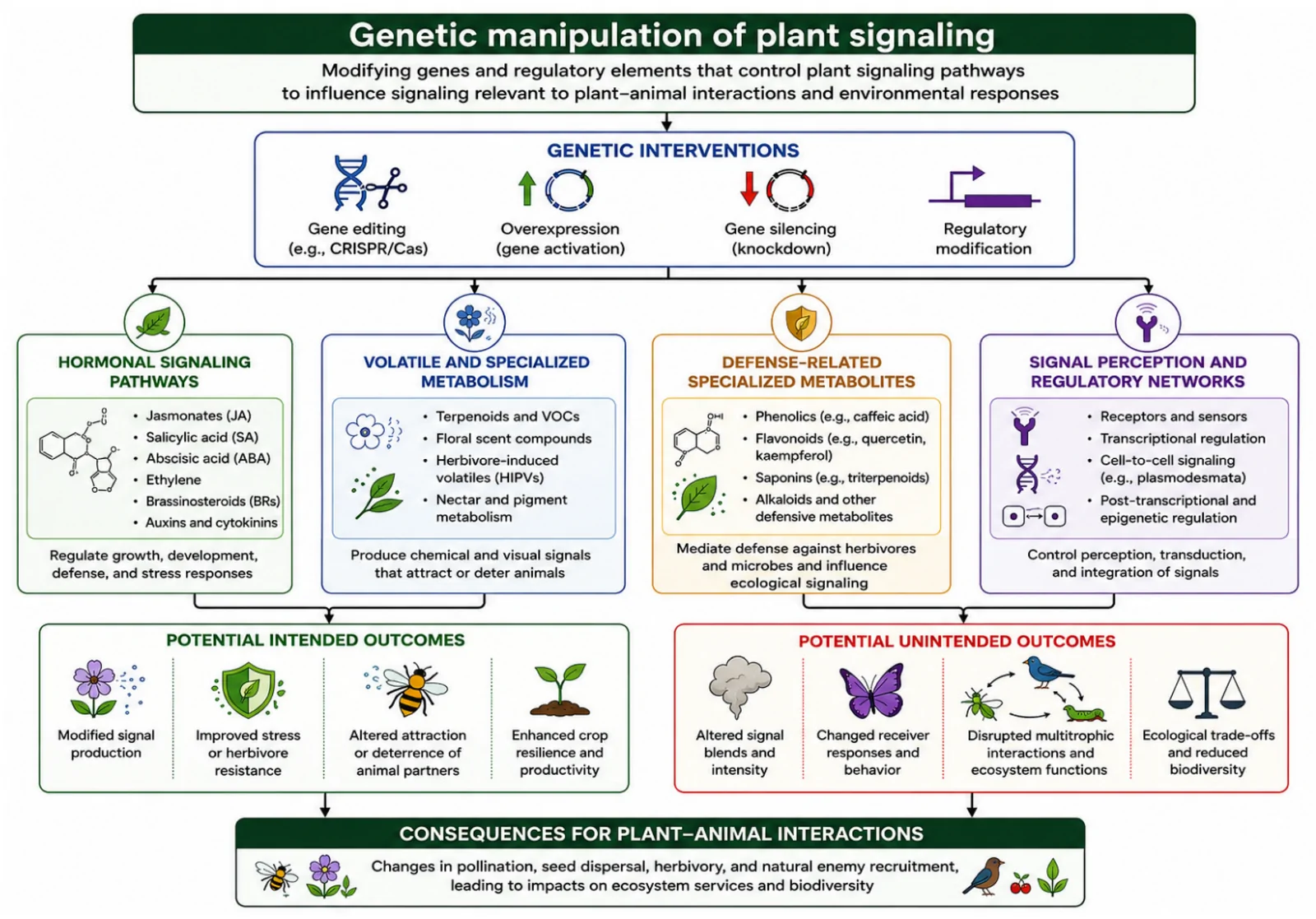
Manipulation of plant signaling pathways and their potential consequences for plant–animal interactions. Intervention strategies, including conventional breeding, transgenic approaches, genome editing, gene silencing, regulatory modification, and exogenous chemical treatments, can alter hormonal signaling, specialized-metabolite pathways, including phenolics and flavonoids, alkaloids, terpenoids and volatile compounds, and other anti-herbivore metabolites, as well as signal-perception and regulatory networks in plants. These interventions may generate intended outcomes, such as altered signal production, improved stress or herbivore resistance, modified attraction or deterrence of animal partners, and enhanced crop resilience. They may also produce unintended effects, including changes in signal blends, receiver responses, non-target interactions, multitrophic relationships, ecosystem functions, and biodiversity. The ecological consequences ultimately depend on the type of intervention, its persistence and pleiotropic effects, and how the resulting changes influence pollination, seed dispersal, herbivory, natural-enemy recruitment, and other plant–animal interactions.

## Data Availability

No new data were created or analyzed in this study. Data sharing is not applicable to this article.
